# Switching-Off Adora2b in Vascular Smooth Muscle Cells Halts the Development of Pulmonary Hypertension

**DOI:** 10.3389/fphys.2018.00555

**Published:** 2018-06-01

**Authors:** Tinne C. J. Mertens, Ankit Hanmandlu, Ly Tu, Carole Phan, Scott D. Collum, Ning-Yuan Chen, Tingting Weng, Jonathan Davies, Chen Liu, Holger K. Eltzschig, Soma S. K. Jyothula, Keshava Rajagopal, Yang Xia, Ashrith Guha, Brian A. Bruckner, Michael R. Blackburn, Christophe Guignabert, Harry Karmouty-Quintana

**Affiliations:** ^1^Department of Biochemistry and Molecular Biology, McGovern Medical School, The University of Texas Health Science Center at Houston, Houston, TX, United States; ^2^Institut National de la Santé et de la Recherche Médicale UMR_S 999, Le Plessis-Robinson, France; ^3^Université Paris-Sud and Université Paris-Saclay, Le Kremlin-Bicêtre, France; ^4^Department of Pediatrics, Baylor College of Medicine, Houston, TX, United States; ^5^Department of Anesthesiology, McGovern Medical School, The University of Texas Health Science Center at Houston, Houston, TX, United States; ^6^Department of Internal Medicine, McGovern Medical School, The University of Texas Health Science Center at Houston, Houston, TX, United States; ^7^Methodist Debakey Heart and Vascular Center, Houston Methodist Hospital, Houston, TX, United States

**Keywords:** Group I PH, Group III PH, hyaluronan, tissue transglutaminase, lung fibrosis, vascular remodeling

## Abstract

**Background:** Pulmonary hypertension (PH) is a devastating and progressive disease characterized by excessive proliferation of pulmonary artery smooth muscle cells (PASMCs) and remodeling of the lung vasculature. Adenosine signaling through the ADORA2B receptor has previously been implicated in disease progression and tissue remodeling in chronic lung disease. In experimental models of PH associated with chronic lung injury, pharmacological or genetic inhibition of ADORA2B improved markers of chronic lung injury and hallmarks of PH. However, the contribution of ADORA2B expression in the PASMC was not fully evaluated.

**Hypothesis:** We hypothesized that adenosine signaling through the ADORA2B receptor in PASMC mediates the development of PH.

**Methods:** PASMCs from controls and patients with idiopathic pulmonary arterial hypertension (iPAH) were characterized for expression levels of all adenosine receptors. Next, we evaluated the development of PH in ADORA2B^f/f^-Transgelin (Tagln)^cre^ mice. These mice or adequate controls were exposed to a combination of SUGEN (SU5416, 20 mg/kg/b.w. IP) and hypoxia (10% O_2_) for 28 days (HX-SU) or to chronic low doses of bleomycin (BLM, 0.035U/kg/b.w. IP). Cardiovascular readouts including right ventricle systolic pressures (RVSPs), Fulton indices and vascular remodeling were determined. Using PASMCs we identified ADORA2B-dependent mediators involved in vascular remodeling. These mediators: IL-6, hyaluronan synthase 2 (HAS2) and tissue transglutaminase (Tgm2) were determined by RT-PCR and validated in our HX-SU and BLM models.

**Results:** Increased levels of ADORA2B were observed in PASMC from iPAH patients. ADORA2B^f/f^-Tagln^cre^ mice were protected from the development of PH following HX-SU or BLM exposure. In the BLM model of PH, ADORA2B^f/f^- Tagln^cre^ mice were not protected from the development of fibrosis. Increased expression of IL-6, HAS2 and Tgm2 was observed in PASMC in an ADORA2B-dependent manner. These mediators were also reduced in ADORA2B^f/f^- Tagln^cre^ mice exposed to HX-SU or BLM.

**Conclusions:** Our studies revealed ADORA2B-dependent increased levels of IL-6, hyaluronan and Tgm2 in PASMC, consistent with reduced levels in ADORA2B^f/f^- Tagln^cre^ mice exposed to HX-SU or BLM. Taken together, our data indicates that ADORA2B on PASMC mediates the development of PH through the induction of IL-6, hyaluronan and Tgm2. These studies point at ADORA2B as a therapeutic target to treat PH.

## Introduction

*Pulmonary Hypertension* (PH) is a condition of the pulmonary vasculature characterized by an mPAP of ≥25 mmHg at rest (Archer et al., 2010). The pathological diagnosis is portrayed as muscularization of previously non-muscular arteries, smooth muscle and endothelial cell proliferation, and the development of vascular lesions (Morrell et al., 2001). PH can be grouped into 5 subsets of hypertension based upon the etiology of the disease. Group I PH, or Pulmonary Arterial Hypertension (PAH), is PH that primarily affects the pre-capillary vasculature of the lungs (Ventetuolo and Klinger, 2012; Hansdottir et al., 2013). Group III PH is associated with chronic lung diseases such as chronic obstructive pulmonary disease (COPD) and idiopathic pulmonary fibrosis (IPF) (Farkas et al., 2011; Fell, 2012; Judge et al., 2012). Group III PH affects between 30 and 80% of patients (Poor et al., 2012; Hansdottir et al., 2013) where it is strongly associated with increased morbidity and mortality (Poor et al., 2012; Ventetuolo and Klinger, 2012; Hansdottir et al., 2013). In the vast majority of cases, PH is not curable. The pathogenesis of PH is poorly understood due to a lack of knowledge of the mechanisms governing its onset and progression. Consequently, research efforts aimed at uncovering the mechanisms involved in disease progression in PH are necessary to stimulate the development of novel therapies for this deadly disorder.

Adenosine is a nucleoside that is elevated following cell injury and stress (Fredholm, 2007). Under conditions of stress or cell injury, ATP is released from the cells and is converted by CD39 and CD73 (CD73 being the rate limiting enzyme) into adenosine (Lennon et al., 1998). Adenosine is then able to bind to one of its four G-protein coupled receptors: the adenosine A1 (ADORA1), A2A (ADORA2A), A2B (ADORA2B), and A3 (ADORA3) receptors (Fredholm et al., 2001). Adenosine is then metabolized extracellularly to inosine by adenosine deaminase (ADA) (Fredholm et al., 2001). In the context of chronic lung disease, increased expression of ADORA2B has been observed in patients with COPD and IPF (Zhou et al., 2010), although it is important to mention that protective effects of ADORA2B have been reported in acute lung injury settings (Karmouty-Quintana et al., 2013b).

In the context of PH, studies performed using explanted lungs from patients with a diagnosis of IPF with and without PH revealed increased expression levels of ADORA2B and enhanced capacity for the generation and accumulation of adenosine levels in patients with Group III PH (Garcia-Morales et al., 2016). Experiments using the ADORA2B antagonist (GS-6201) or *full* Adora2b knock-out mice revealed that genetic deletion or pharmacological inhibition of Adora2b subdued both the fibrotic deposition and the development of PH in a mouse model of chronic bleomycin (BLM)-induced lung fibrosis and PH (Karmouty-Quintana et al., 2012). Further studies revealed that conditional deletion of Adora2b from the myeloid lineage resulted in a reduction in fibrotic deposition and the absence of hallmarks of PH, including thickening of the vascular wall and elevated right ventricle systolic pressure (RVSP), in mice with conditional deletion of Adora2b in myeloid cells (Karmouty-Quintana et al., 2015). In experiments using pulmonary artery smooth muscle cells (PASMC), studies have shown that activation of ADORA2B can lead to increased expression of hyaluronan synthase (HAS) isozymes 1 and 2, enhancing levels of hyaluronan (Karmouty-Quintana et al., 2013a), the major glycosaminoglycan in the lungs that when fragmented has been implicated in modulating PH and lung fibrosis (Karmouty-Quintana et al., 2013a; Collum et al., 2017b). However, expression levels of CD39, CD73, ADA, and adenosine receptors in PAH has not yet been evaluated. In addition, the effects of conditional deletion of ADORA2B from vascular smooth muscle cells on the development of PH have not yet been determined. Here, we have assessed expression levels of mediators involved in the generation and metabolism of adenosine in PAH and evaluated the effects of conditional deletion of ADORA2B in vascular smooth muscle cells using the transgelin (Tagln) promoter, also known as smooth muscle protein 22-alpha promoter. In these *in vivo* experiments, mice were exposed to two distinct models of PH: the chronic hypoxia-SUGEN (HX-SU) model of PH and the BLM model of lung fibrosis and PH. In addition, we performed cell culture studies using human isolated PASMCs to identify ADORA2B-mediated mechanisms leading to PH.

## Materials and methods

### Isolation of human pulmonary artery smooth muscle cells (PASMCs)

Human PASMCs were isolated and cultured as previously described (Guignabert et al., 2005; Huertas et al., 2015). To identify PASMCs, we examined cultured cells for expression of muscle specific contractile and cytoskeletal proteins including smooth-muscle α actin, desmin, and Tagln. Cells were used between passages 3 and 6. A minimum N of 5 was used for all experiments using PASMCs. The clinical data from the donors were the PASMCs were isolated for western blots is available in Supplementary Table 1. The clinical data for PASMCs used for RT-PCR experiments can be found in the following publication: (Huertas et al., 2015, Table 2). Our studies using human material was reviewed by an institutional review board (IRB): HSC-MS-08-0354.

### Cell culture experiments

Primary human pulmonary artery smooth muscle cells (PASMCs) were plated at 3,000 cells/cm^2^ and grown in DMEM containing 10% FBS and antibiotics until 70–80% confluence. Next, PASMCs were serum starved overnight followed by 72 h exposure to normoxia or 2% O_2_ (hypoxia) using a modular incubator chamber (Billups-Rothenberg, San Diego, CA, USA). PASMC were exposed for 72 h in combination with normoxia or hypoxia to the ADORA2B agonist BAY 60-6583 (10 μM) with or without the ADORA2B antagonist GS-6201 (100 nM) (both Tocris Bioscience, Bristol, UK). DMSO was used as a solvent control.

### Animals

Adora2b^*f*/*f*^-Tagln^*Cre*^ and Tagln^*Cre*^ mice on the C57/Bl6 background were used for all experiments. Animals were mated and genotyped as described previously (Zimmerman et al., 2013). All mice were housed in ventilated cages equipped with microisolator lids and maintained under strict containment protocols. N of 5 was used for all experimental groups (except for western blots). Mice were randomized to group treatment using a random number generator using www.graphpad.com/quickcalcs. For sample analysis, all mice were ear-tagged and researchers were blinded to the treatment group. In cases where data was normalized, this was performed to control for unwanted sources of variation. Mice were housed 5 per cage and were provided with variable free paper bedding (Pure-o'Cel The Andersons, Inc. Maumee, Ohio, USA) and Nestlets^TM^ provided by Ancare (Bellmore, NY). The red mouse loft (Tecniplast, Buguggiate, Italy) was provided as amusement to all mouse cages. Mice were kept at an ambient temperature of 22°C and in a 12 h dark/light cycle. Animal care was in accordance with institutional and NIH guidelines. All studies were reviewed and approved by the University of Texas Health Science Center at Houston Animal Welfare Committee. Following consultation with a statistician, our experimental N number was set to 5 based on a power analysis (*F*-tests ANOVA:One Way) with the following criteria: alpha error: 0.05, Power: 0.95, number of groups 4, f:1.189. The Power and *f*-values were calculated *post-hoc* using previous data generated by our lab, including pulseox values, right ventricle systolic pressures, Fulton Indices and gene expression data for fibrotic markers. G^*^Power 3.1.9.2 Universität Düsseldorf, Germany was used for all the analysis.

In order to verify that Adora2b was depleted in PASMCs; primary cultures of pulmonary artery smooth muscle cells (PASMC) from mice were isolated as previously described (Lee et al., 2013) with some minor alterations. A mixture of 0.5% (w/v) agarose + 0.5% iron particles in DMEM containing antibiotics was infused through the right ventricle, resulting in lodging of 0.2 μM iron particles in PAs. The lungs were then inflated with 1% (w/v) agarose in DMEM containing antibiotics, removed, and dissociated. The iron-containing vessels were pulled down with a magnet, treated with 0.2% (w/v) collagenase, Type 2 (Worthington, Lakewood, NY, USA), and for 45 min. The resulting PASMC were maintained in DMEM media with 25 mM HEPES, 10% fetal bovine serum (FBS) and antibiotics at 37°C in a humidified atmosphere with 5% CO^2^. After isolation and reaching 80–90% confluence, cells were lysed for RNA and protein extraction. Depletion of Adora2b was assessed on isolated primary pulmonary artery smooth muscle cells (PASMCs) from Tagln^*Cre*^ and Adora2b^*f*/*f*^-Tagln^*Cre*^ mice where Cre expression was also determined (Supplementary Figure 1).

### Experimental design

In experiments involving chronic exposure to hypoxia-SUGEN (HX-SU), mice were placed in open cages inside a specifically designed chamber (A-Chamber, Biospherix, Lacona, NY) and exposed to 10% oxygen for 4 weeks. The oxygen levels were maintained using the Oxygen regulator from OKO Labs (Pozzuoli, NA, Italy). In our model, SUGEN (SU5416; Tocris, Bristol, BS11 9QD United Kingdom) was dissolved in a solution of 10% Kolliphor® HS 15 (Macrogol (15)-hydroxystearate, Sigma Aldrich, St Louis. MO) and administered once weekly via the intra-peritoneal route (20 mg/kg/b.w. IP). Control mice were housed in the same room and were exposed to ambient air (normoxia) for 4 weeks and received the vehicle for SUGEN (10% Kolliphor® once weekly IP). In our bleomycin (BLM) model of chronic lung injury, mice were exposed to BLM (0.035 U/kg/b.w. IP) or vehicle (PBS) twice weekly for 4 weeks as previously described (Karmouty-Quintana et al., 2012). Mice were euthanized on day 28 in the HX-SU model and on day 33 for the BLM model for histological analysis after physiological measurements were performed. A minimum N of 5 was used for experimental groups.

### Hemodynamic measurements: right ventricle systolic pressure (RVSP), heart rate, and RV hypertrophy

This procedure was performed as previously described (Karmouty-Quintana et al., 2012). Briefly, mice were given 0.75 mg/g of 2.5% Avertin (a mixture of tert-amyl alcohol and 2-2-2 Tribromoethanol, Sigma Aldrich, ST. Louis, MO) to induce a surgical plane of anesthesia. Mice were placed on a heated pad (Deltaphase Isothermal pad model 39; Braintree Scientific, Braintree, MA) and secured with surgical tape. Mice were then tracheotomised with a 19G blunt needle (BRICO, Dayton, NJ) and attached to a small animal ventilator (MiniVent, Hugo-Sachs Elektronik, March-Hugstetten, Germany) and ventilated at a stroke volume of 250 μl at 60 strokes per minute. The surgical site was viewed using a surgical microscope (SMZ-2B, Nikon, Tokyo, Japan). An incision of ~1 cm in length was made just below the xiphoid process. An alm retractor (ALM-112, Braintree Scientific, Braintree, MA) was used to expose the abdominal cavity to visualize the diaphragm and the liver. An incision was then made on the diaphragm to expose the heart and the pericardium was removed. The right ventricle was then identified and a puncture was made with a 27G needle. A 1 French pressure catheter (SPR-1000, Millar Instruments, Houston, TX) was then inserted through the puncture. The heart rate results were continuously recorded using a Powerlab 8-SP A/D (AD Instruments) converter, acquired at 1000 Hz. All RVSP results were recorded to a PC utilizing Chart5.3 software. After completion of the measurements, blood was collected and the lungs were excised and flash frozen in liquid nitrogen for RNA extraction. The heart was excised and the atria were removed. The right ventricle was then surgically removed and the dry weights of the RV were used to determine the Fulton Index: extent of RV-hypertrophy using the weight of the left ventricle and septum to normalize the data (RV/LV+S).

### Histology, immunohistochemistry, and western blots

Mouse lungs were inflated with 10% buffered formalin at 25 cm of water and fixed at 4°C overnight. Lungs were dehydrated in ethanol gradients and embedded in paraffin, and 5-μm tissue sections were collected on microscope slides and stained with Masson's trichrome (EM Science, Gibbstown, NJ) according to manufacturer's instructions.

Immunohistochemistry was performed on 5 μm sections cut from formalin-fixed, paraffin-embedded lungs. Sections were rehydrated through graded ethanol to water, antigen retrieval was performed using a solution of 10 mM citric acid and heated for 2 min for 3 intervals at high power using a microwave, endogenous peroxidase and alkaline phosphatase were inactivated using BLOXALL (Vector Labs, Burlingame, CA) and 2.5% normal horse serum (Vector Labs) was used as a blocking solution prior to incubation with the primary antibody. Following overnight incubation with the primary antibody, sections were treated with the ImmPRESS polymer detection kits for alkaline phosphatase or horse-radish peroxidase (Vector Labs) based on the host of the primary antibody and development method. Slides were incubated with primary antibodies (see Supplementary Table 1). Sections were developed with VIP-HRP Substrate Kit or Vector Blue/Red or BCIP/NBT Alkaline Phosphatase Substrate Kits (Vector Laboratories). Slides were mounted using cytoseal or mountiung medium containing DAPI (Sigma Aldrich).

For Western blots, protein from lung tissue lysates or PASMCs was extracted with RIPA buffer (Thermo Scientific, Rockford, IL) containing 1 mM of protease and phosphatase inhibitor (Sigma Aldrich, St Louis, MO). Thirty microgram of protein per sample was loaded onto 4–12% Mini-Protean TGX gels (Bio-Rad, Hercules, CA) for electrophoresis and then transferred on polyvinylidene difluoride (PVDF) membranes (0.45 μm, GE Healthcare Piscataway, NJ). Membranes were then blocked in 5% Milk (Bio-Rad) for 1 h at room temperature and then incubated with the appropriate primary antibody overnight (see Supplementary Table 2). Secondary antibodies and an ECL kit (GE Healthcare) were applied for generating chemiluminescent signals. Isotype control images for all IHC staining is available in Supplementary Figure 2.

### Morphometry

Muscularized arterioles of the lung parenchyma were observed under 20x magnification and noted as being different from both airways and non-muscularized arterioles. Muscularized arterioles were then photographed under 40x magnifications. Micropictographs were then analyzed using Image Pro-Plus software (MediaCybernetics Inc, Bethesda, MD). In short, the overall area of the muscularized portion was measured for each arteriole. To account for size, the largest diameter for each arteriole was also measured. The area of the arteriole was then divided by the largest diameter to give a relative measurement of muscularization. To determine fibrotic deposition, lung sections were stained for Masson's Tri-chrome and analyzed using a modified Ashcroft scale optimized for mouse lung sections (Hubner et al., 2008). Ten images per animal were analyzed by 3 individuals blinded to group status.

### RT-PCR

Total RNA was isolated from frozen lung tissue using Trizol reagent (Life Technologies). RNA samples were then DNase treated (ArticZymes, Tromso, Norway) and subjected to quantitative real-time RT-PCR. The specific primers used is available in Supplementary Table 3. Data is presented as mean normalized transcript levels using the comparative Ct method (2ΔΔCt).

### HPLC

Adenosine levels in bronchoalveolar lavage fluid (BALf) were measured as previously described (Wakamiya et al., 1995). Briefly, to measure the nucleoside levels in BALF, mice were anesthetized with 2.5% avertin and the lungs were lavaged 4 times with 0.3 ml PBS containing 2 μM dipyridamole (Sigma-Aldrich, St. Louis, MO, USA) and 5 μM ADA-inhibitor deoxycoformycin (dCF, R&D Systems Inc, Minneapolis, MN, USA), which pooled 1.0 ml BALF. The BALF was then centrifuged to remove cells and debris. To measure nucleoside levels, 200 μl BALF supernatant were loaded to the HPLC meter per reading and the flow rate was set at 1 ml/min. The representative peaks were identified and quantitated by running known external standard curves.

### Statistical analysis

All analyses were blinded to the experimenter. A two-way analysis of variance (ANOVA) with a Tukey *post hoc* test was performed for all experiments with more than 2 groups. For experiments that consisted of two groups, an un-paired two-tailed Students *t*-test with a Welch correction was performed. Categorical data was analyzed using a Chi-Squared calculation. Statistical significance was defined as *P* ≤ 0.05 by use of GraphPad Prism version 5 (GraphPad Software, La Jolla, CA). Densitometry analysis from Western blots were performed using ImageJ (National Institutes of Health, Bethesda, Maryland, USA). The Grubbs' test was used to detect outliers.

## Results

### Enhanced adenosinergic system in idiopathic pulmonary arterial hypertension (iPAH)

**Figure 1:**
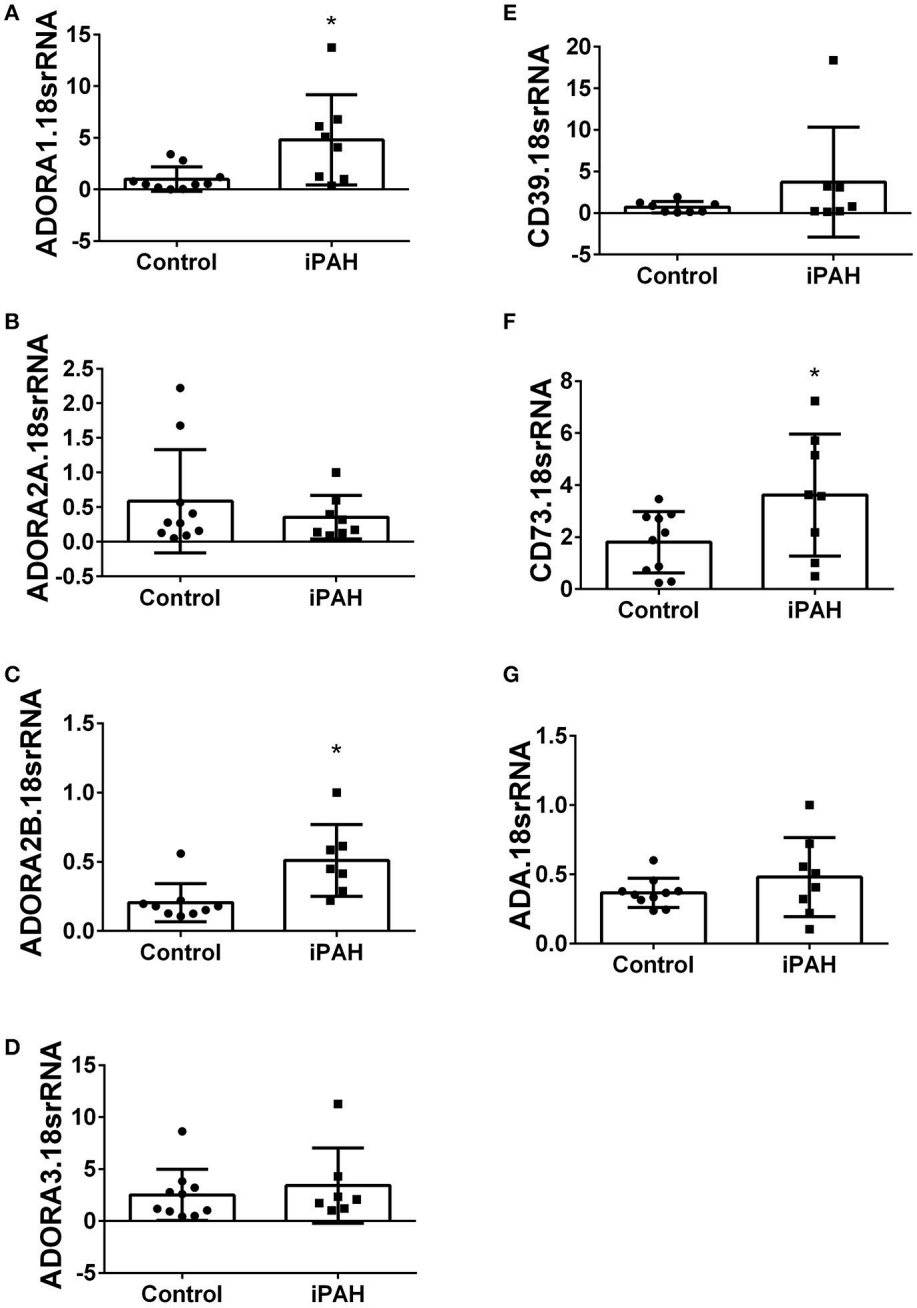
Expression levels of adenosine receptors and genes associated with adenosine synthesis in pulmonary artery smooth muscle cells (PASMCs) derived from controls and idiopathic pulmonary hypertension (iPAH) lung tissue. ADORA1 **(A)**; ADORA2A **(B)**; ADORA2B **(C)**; ADORA3 **(D)**; CD39 **(E)**; CD73 **(F)**; and adenosine deaminase **(**ADA, **G**). Results are presented as means ± SE, *N* = 10 (control) or 8 (iPAH) and normalized to the expression of 18srRNA. ^*^P < 0.05 refers to comparisons between control vs. iPAH groups.

Using isolated PASMCs from normal controls or patients with a diagnosis of idiopathic pulmonary arterial hypertension (iPAH), we first profiled the expression levels of adenosine receptors (Figures 1A–D). In these experiments, we identified increased transcript levels of ADORA1 (Figure 1A) and ADORA2B (Figure 1C) but not ADORA2A or ADORA3 (Figures 1B,D). Subsequently, we determined expression levels of CD39 and CD73, key enzymes for the catabolism of ATP to adenosine. These experiments did not show significant changes for CD39 in iPAH PASMCs compared to controls (Figure 1E). CD73, the rate-limiting enzyme, was significantly elevated in iPAH vs. control PASMCs (Figure 1F); yet no changes in ADA expression were seen (Figure 1G). Expression levels of ADA did not appear altered in iPAH vs. control PASMCs.

**Figure 2:**
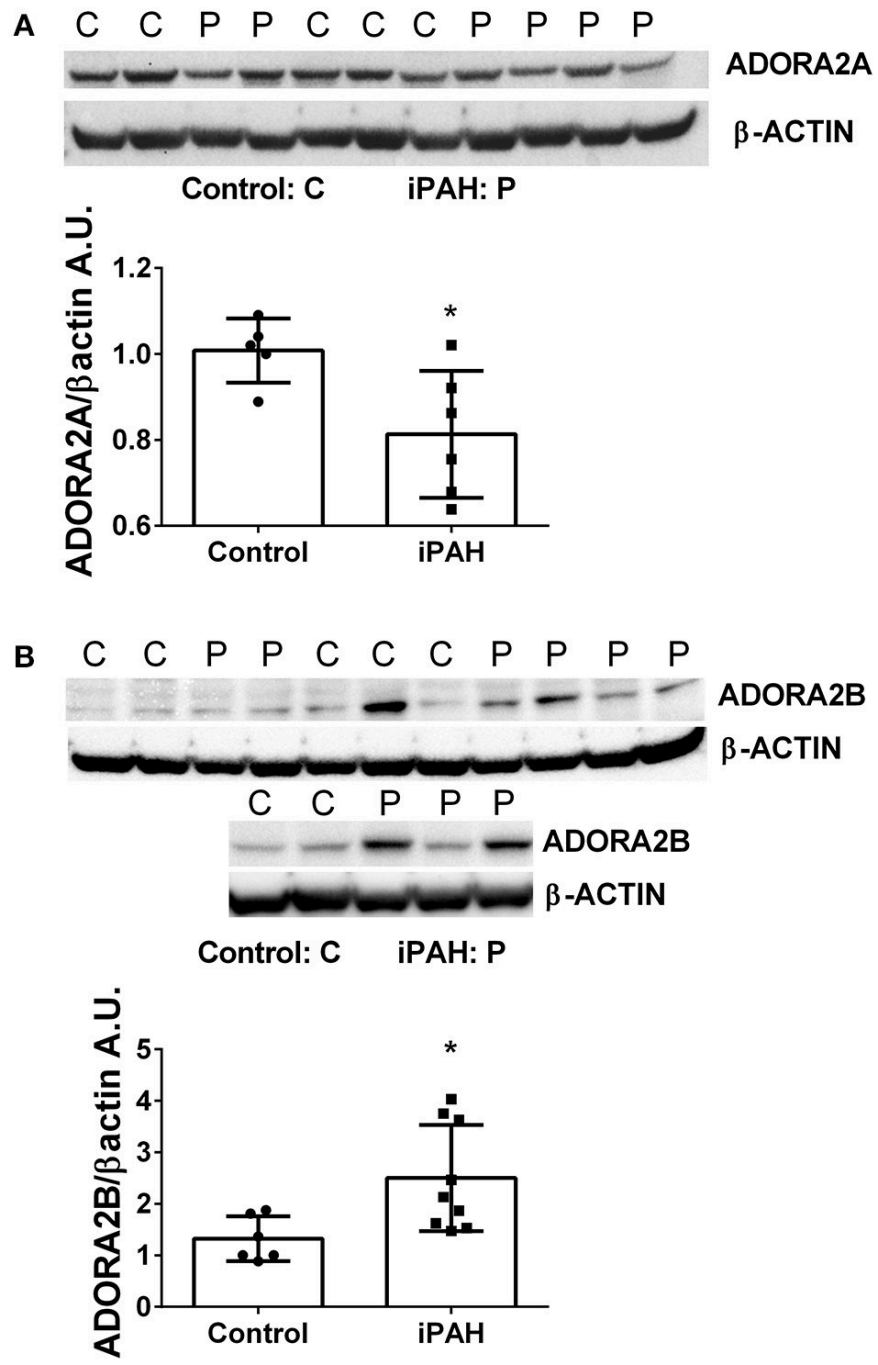
Protein levels of adenosine receptors in pulmonary artery smooth muscle cells (PASMCs) derived from controls and idiopathic pulmonary hypertension (iPAH). Protein expression levels and quantification of ADORA2A **(A)** and ADORA2B **(B)** levels in isolated PASMCs from control or iPAH patients. β-ACTIN was used to normalize expression levels of ADORA2A and ADORA2B, blots are shown. ^*^P < 0.05 refers to comparisons between control vs. iPAH groups. *N* = 7 Control, *N* = 6 iPAH for **(A)** and *N* = 6 Control, *N* = 9 iPAH for **(B)**. Lane 6 from **(B)** was excluded from the analysis as it was determined as an outlier by the Grubb's Test.

We next used Western blots to validate expression levels of ADORA2A and ADORA2B. Remarkably, iPAH PASMCs showed attenuated levels of ADORA2A but elevated ADORA2B (Figures 2A,B); the latter consistent with transcript levels for ADORA2B. Collectively, these data point at enhanced capacity for the generation of adenosine in PASMCs from iPAH patients in addition to increased altered expression of adenosine receptors leading to increased ADORA2B, but reduced ADORA2A. These results point at altered adenosine receptor expression in human PASMCs from patients with iPAH.

### Enhanced adenosinergic system in mice exposed to hypoxia-sugen (HX-SU) or bleomycin (BLM)

**Figure 3:**
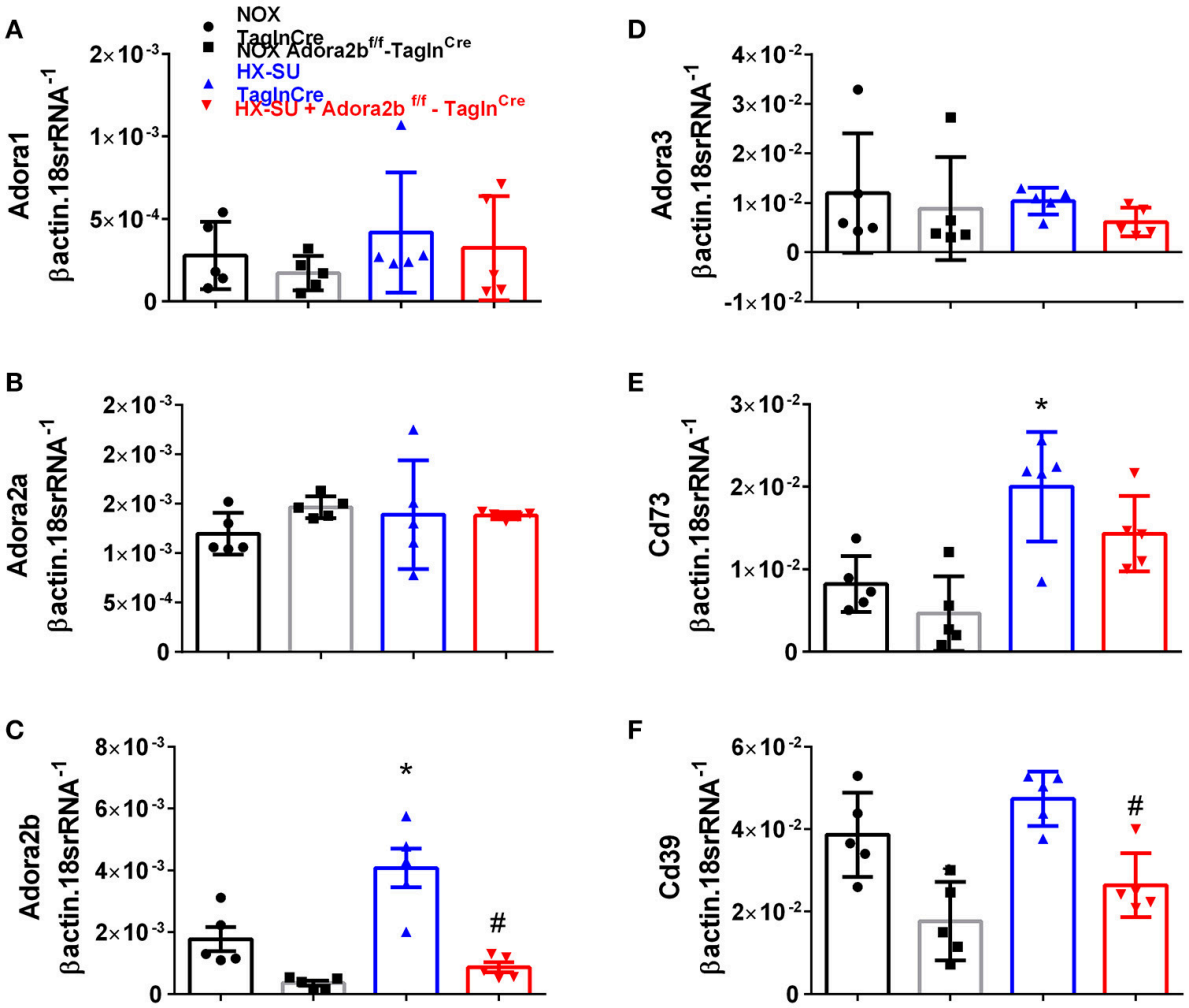
Expression levels of adenosine receptors and genes associated with adenosine synthesis in TaglnCre and Adora2b^f/f-^Tagln^Cre^ mice exposed to hypoxia-SUGEN (HX-SU) or normoxia (NOX). All analyses were performed on day 28 of HX-SU and NOX exposure. Adora1 **(A)**; Adora2a **(B)**; Adora2b **(C)**; Adora3 **(D)**; Cd73 **(E)**; Cd39 **(F)**; expression levels measured by RT-PCR and normalized to the Geo mean of expression levels of βactin and 18srRNA from NOX-Tagln^Cre^ mice (black circles and bar outline); NOX- Adora2b^f/f-^Tagln^Cre^ mice (black squares and gray bar outline); HX-SU-Tagln^Cre^ mice (blue triangles and bar outline); HX-SU-Adora2b^f/f-^Tagln^Cre^ mice (red triangles and bar outline). Significant values: ^*^P < 0.05 refer to comparisons between Tagln^Cre^ HX-SU and Tagln^Cre^ NOX treatment groups. ^#^*P* < 0.05, are for comparisons between Tagln^Cre^ HX-SU and Adora2b^f/f-^Tagln^Cre^ + HX-SU treatment groups. *N* = 5 for all groups.

In order to model pulmonary hypertension, we exposed mice for 4 weeks to hypoxia and treated them once weekly with SUGEN (SU5416, 20 mg/kg bw). This protocol was adapted from earlier studies (Ciuclan et al., 2011). RT-PCR from flash frozen lungs revealed no significant changes in Adora1 or Adora2a expression levels between mice exposed to HX-SU or normoxia (NOX) in Tagln^Cre^ or Adora2b^f/f^-Tagln^Cre^ mice (Figures 3A,B). However, consistent with our observations in iPAH PASMCs, we report increased Adora2b transcripts in HX-SU–exposed Tagln^Cre^ mice compared to NOX exposed Tagln^Cre^ mice which were attenuated in HX-SU exposed Adora2b^f/f^-Tagln^Cre^ mice (Figure 3C). No significant differences were observed in Adora3 expression levels between treatment groups (Figure 3D). Interestingly, increased expression levels of CD73 were observed between HX-SU–exposed Tagln^Cre^ compared to NOX exposed Tagln^Cre^ mice, albeit no differences between HX-SU exposed Adora2b^f/f^-Tagln^Cre^ and HX-SU Tagln^Cre^ mice (Figure 3E). CD39 expression analysis revealed reduced levels in Adora2b^f/f^-Tagln^Cre^ mice exposed to normoxia or HX-SU compared to Adora2b competent mice (Figure 3F). In summary HX-SU exposed Adora2b^f/f^-Tagln^Cre^ mice showed reduced expression levels of Adora2b and CD39 compared to HX-SU exposed Tagln^Cre^ mice (Figures 3C,F).

**Figure 4:**
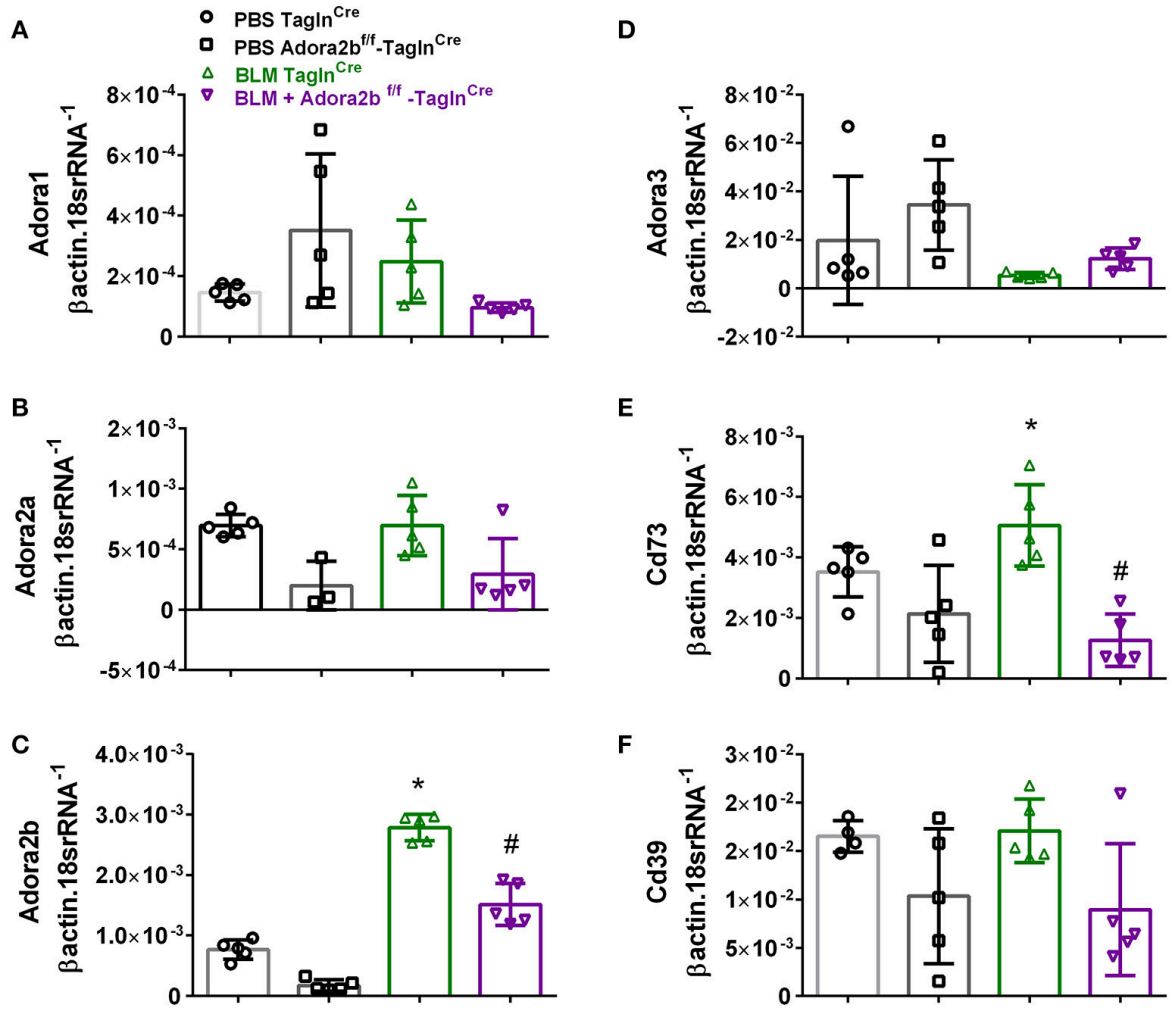
Expression levels of adenosine receptors and genes associated with adenosine synthesis in TaglnCre and Adora2b^f/f−^Tagln^Cre^ mice exposed to bleomycin (BLM) or vehicle phosphate-buffered saline (PBS). All analyses were performed on day 33 of PBS or BLM exposure. Adora1 **(A)**; Adora2a **(B)**; Adora2b **(C)**; Adora3 **(D)**; Cd73 **(E)**; Cd39 **(F)**; expression levels measured by RT-PCR and normalized to the Geo mean of expression levels of βactin and 18srRNA from PBS-Tagln^Cre^ mice (black circles and bar outline); PBS- Adora2b^f/f−^Tagln^Cre^ mice (black squares and bar outline); BLM-Tagln^Cre^ mice (green triangles and bar outline); BLM-Adora2b^f/f−^Tagln^Cre^ mice (magenta triangles and bar outline). Significant values: ^*^P < 0.05 refer to comparisons between Tagln^Cre^ BLM and Tagln^Cre^ PBS treatment groups. ^#^*P* < 0.05, are for comparisons between Tagln^Cre^ BLM and Adora2b^f/f^-Tagln^Cre^ + BLM treatment groups. *N* = 5 for all groups.

These changes were consistent with mice chronically exposed to low doses of BLM IP where no significant changes in Adora1, Adora2a, or Adora3 expression levels were observed between Adora2b^f/f^-Tagln^Cre^ or Tagln^Cre^ mice expose to either BLM or PBS (Figures 4A,B,D). In line with our HX-SU exposed mice and with our previous studies using BLM-treated mice (Karmouty-Quintana et al., 2012), increased levels of Adora2b were observed in BLM-exposed Tagln^Cre^ mice compared to PBS-treated mice (Figure 4C). Consistent with the depletion of Adora2b using the Tagln^Cre^ promoter, we report reduced expression levels of Adora2b in Adora2b^f/f^-Tagln^Cre^ mice exposed to either BLM or PBS (Figure 4C). Expression levels of CD73 demonstrated increased levels in the BLM- Tagln^Cre^ group (Figure 4E). No significant changes in CD39 were reported between treatment groups (Figure 4F). BLM- exposed Adora2b^f/f^-Tagln^Cre^ mice showed reduced expression levels of Adora2b and CD73 compared to HX-SU exposed Tagln^Cre^ mice (Figures 4C,E). These results demonstrate Adora2b expression is augmented in Tagln^Cre^ mice following BLM-exposure, consistent with increased CD73 expression. Interestingly, expression levels of Adora2b were only partially attenuated in BLM-exposed Adora2b^f/f^-Tagln^Cre^ mice. These results likely reflect Adora2b expression in non-Tagln^Cre^-expressing cells.

### Vascular deletion of Adora2B prevents the development of hypoxia-SUGEN (HX-SU)-induced pulmonary hypertension (PH)

**Figure 5:**
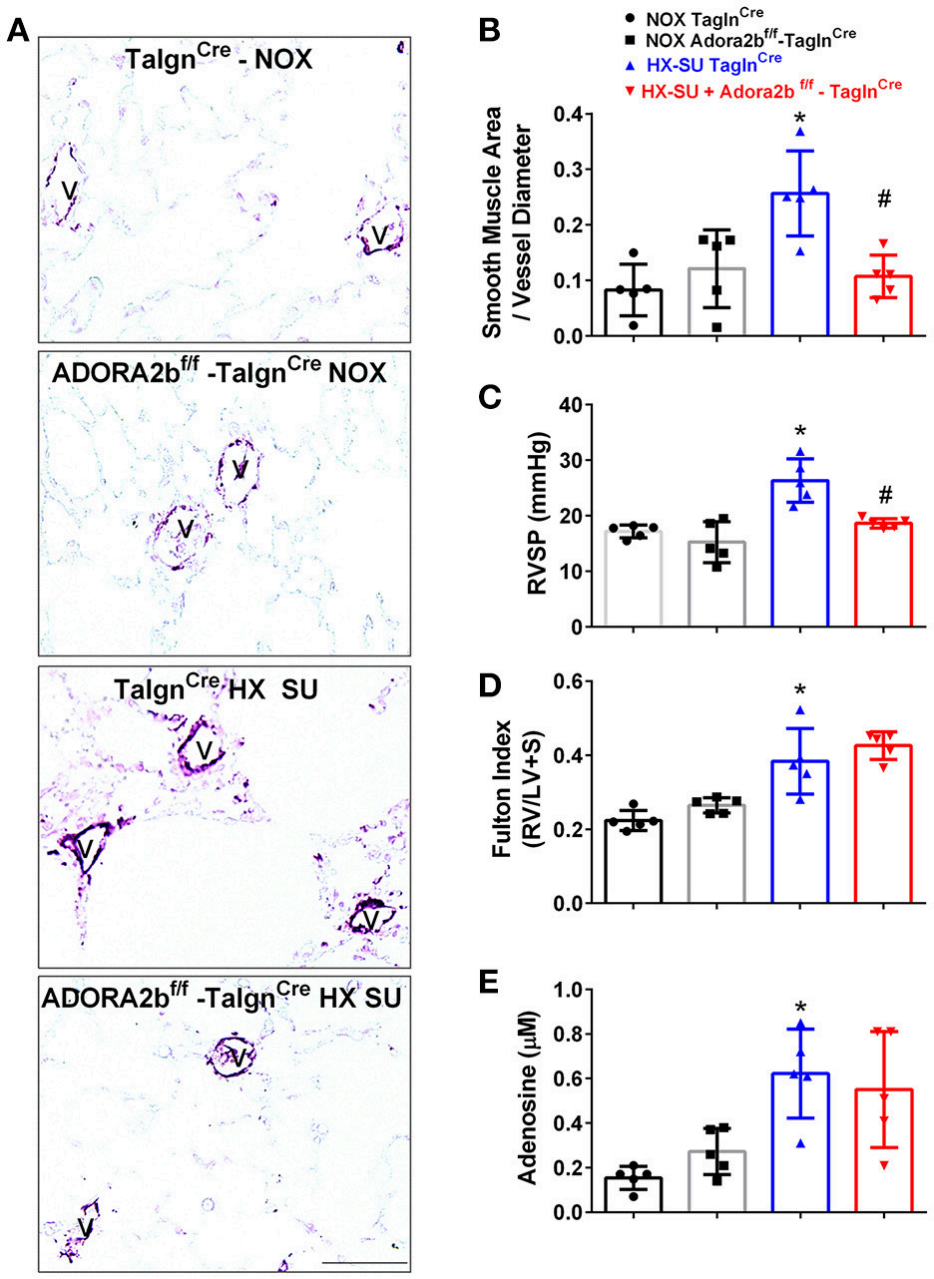
Cardiovascular physiology in TaglnCre and Adora2b^f/f-^Tagln^Cre^ mice exposed to hypoxia-SUGEN (HX-SU) or normoxia (NOX). All analyses were performed on day 28 of HX-SU and NOX exposure. **(A)** Representative immunohistochemistry for alpha smooth muscle actin (αSMA, violet-purple signals) vessels are demarked by a **V** from Tagln^Cre^ and Adora2b^f/f-^Tagln^Cre^ mice exposed to NOX or HX-SU. Morphometric evaluation of αSMA deposition from 5-7 vessels for each mouse **(B)**; Right ventricle systolic pressure (RVSP) **(C)**; Fulton Index ratio determined by the measure of right ventricle and left ventricle with septum **(D)**; and broncho-alveolar lavage fluid (BALF) adenosine levels measured by HPLC **(E)**; from NOX-Tagln^Cre^ mice (black circles and bar outline); NOX- Adora2b^f/f-^Tagln^Cre^ mice (black squares and gray bar outline); HX-SU-Tagln^Cre^ mice (blue triangles and bar outline); HX-SU-Adora2b^f/f-^Tagln^Cre^ mice (red triangles and bar outline). Significant values: ^*^P < 0.05 refer to comparisons between Tagln^Cre^ HX-SU and Tagln^Cre^ NOX treatment groups. ^#^*P* < 0.05, are for comparisons between Tagln^Cre^ HX-SU and Adora2b^f/f^-Tagln^Cre^ + HX-SU treatment groups. *N* = 5 for all groups.

We next examined the role of vascular deletion of Adora2b in the development of HX-SU-induced PH. In these experiments, Tagln^Cre^ mice exposed to HX-SU presented with increased α-smooth muscle actin (αSMA) deposition, consistent with vascular remodeling, compared to Tagln^Cre^ and Adora2b^f/f^-Tagln^Cre^ exposed to normoxia (Figure 5A). HX-SU-exposed Adora2b^f/f^-Tagln^Cre^ mice showed reduced vascular αSMA deposition compared to HX-SU exposed Tagln^Cre^ mice (Figure 5A). These histological observations were consistent with morphometric analyses showing evidence of vascular remodeling in HX-SU-Tagln^Cre^ mice compared to mice exposed to normoxia. Adora2b^f/f^-Tagln^Cre^ mice exposed to HX-SU showed a significant reduction in vascular remodeling compared to HX-SU exposed Tagln^Cre^ mice (Figure 5B). RVSP measurements revealed increased pressures in HX-SU exposed Tagln^Cre^ mice compared to mice exposed to normoxia. HX-SU exposed Adora2b^f/f^-Tagln^Cre^ mice showed reduced RVSP levels in comparison with HX-SU exposed Tagln^Cre^ mice (Figure 5C). We next examined the extent of right ventricle hypertrophy (RVH) using the Fulton index. Here we report an increased Fulton index in Tagln^Cre^ mice exposed to HX-SU compared to normoxia-exposed mice. Remarkably, HX-SU-Adora2b^f/f^-Tagln^Cre^ mice did not show a reduced extent of RVH compared to HX-SU Tagln^Cre^ mice (Figure 5D). Adenosine levels in BALF revealed increased levels in Tagln^Cre^ mice exposed to HX-SU compared to mice exposed to normoxia that remained elevated in HX-SU Adora2b^f/f^-Tagln^Cre^ mice (Figure 5E). These observations are in line with RT-PCR data showing increased CD73 levels in HX-SU Tagln^Cre^ mice that are maintained in conditional KO mice lacking Adora2b. Taken together, these results demonstrate that conditional deletion of Adora2b from vascular smooth muscle cells attenuated the development of PH in mice despite increased levels of adenosine remaining unchanged. Interestingly, in this model, the Fulton index remained elevated in HX-SU exposed Adora2b^f/f^-Tagln^Cre^ mice.

### Vascular deletion of Adora2B prevents the development of bleomycin (BLM)-induced pulmonary hypertension (PH)

**Figure 6:**
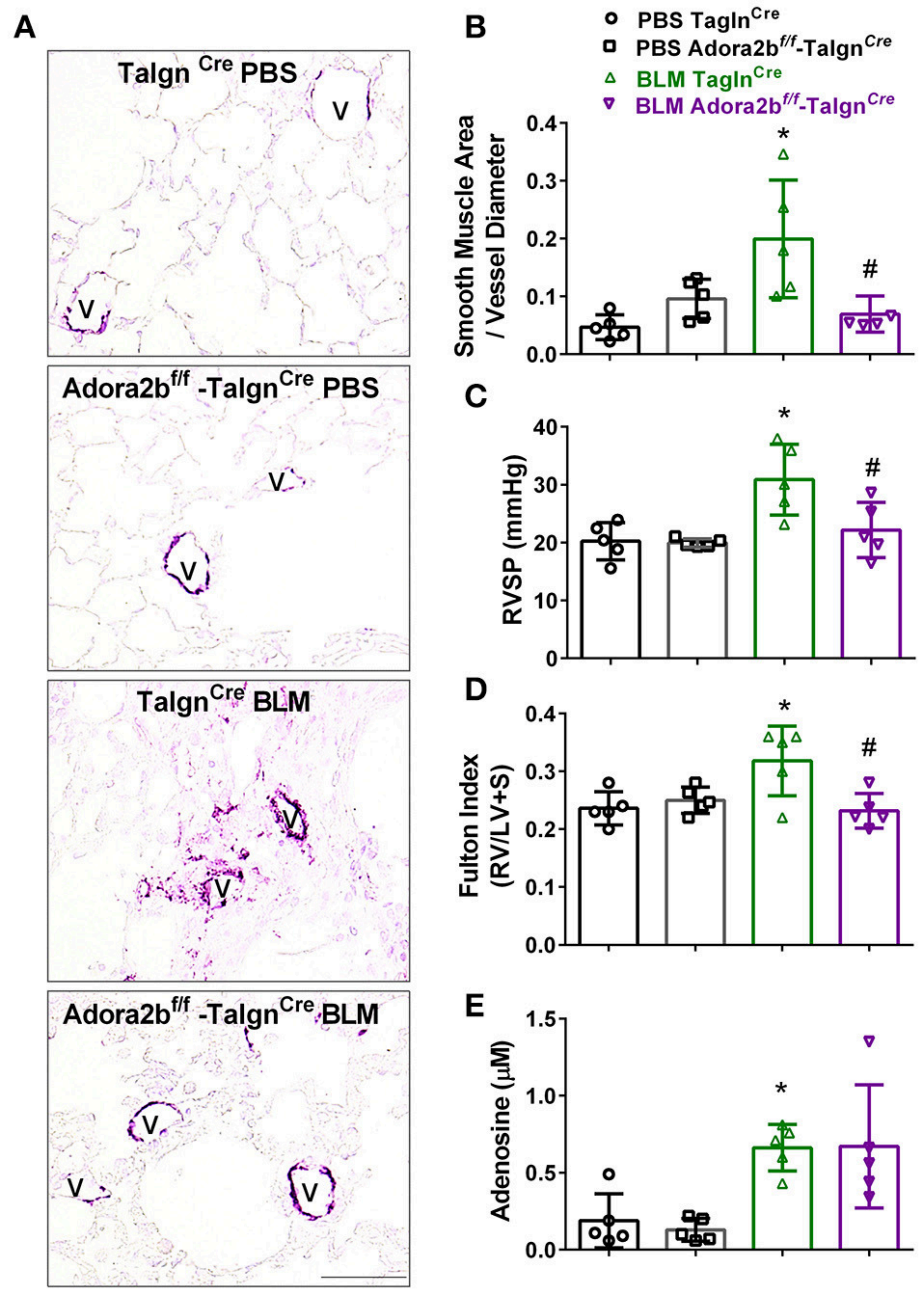
Cardiovascular physiology in Tagln^Cre^ and Adora2b^f/f−^Tagln^Cre^ mice exposed to bleomycin (BLM) or phosphate-buffered saline vehicle (PBS). All analyses were performed on day 33 of BLM and PBS exposure. **(A)** Representative immunohistochemistry for alpha smooth muscle actin (αSMA, violet-purple signals) vessels are demarked by a **V** from Tagln^Cre^ and Adora2b^f/f−^Tagln^Cre^ mice exposed to PBS or BLM. Morphometric evaluation of αSMA deposition from 5 to 7 vessels for each mouse **(B)**; Right ventricle systolic pressure (RVSP) **(C)**; Fulton Index ratio determined by the measure of right ventricle and left ventricle with septum **(D)**; and broncho-alveolar lavage fluid (BALF) adenosine levels measured by HPLC **(E)**; from PBS-Tagln^Cre^ mice (black circles and bar outline); PBS- Adora2b^f/f−^Tagln^Cre^ mice (black squares and bar outline); BLM-Tagln^Cre^ mice (green triangles and bar outline); BLM-Adora2b^f/f−^Tagln^Cre^ mice (magenta triangles and bar outline). Significant values: ^*^P < 0.05 refer to comparisons between Tagln^Cre^ BLM and Tagln^Cre^ PBS treatment groups. ^#^*P* < 0.05, are for comparisons between Tagln^Cre^ BLM and Adora2b^f/f^-Tagln^Cre^ + BLM treatment groups. *N* = 5 for all groups.

We next used our BLM-induced model of lung fibrosis and PH to evaluate the effect of conditional deletion of Adora2b in vascular smooth muscle cells in a model mimicking features of Group III PH. Immunohistochemistry for αSMA revealed increased vascular αSMA deposition in BLM exposed Tagln^Cre^ mice compared to PBS-exposed Tagln^Cre^ mice, which appeared to be attenuated in the BLM-treated Adora2b^f/f^-Tagln^Cre^ group (Figure 6A). No significant changes in αSMA deposition were observed in PBS-exposed Adora2b^f/f^-Tagln^Cre^ mice compared to Tagln^Cre^ mice receiving PBS (Figure 6A). Morphometric determination of vascular remodeling showed thickening of the vascular wall in BLM-treated Tagln^Cre^ mice compared to mice receiving PBS. BLM-treated Adora2b^f/f^-Tagln^Cre^ mice showed significantly reduced vascular remodeling, observed histologically and morphometrically, compared to BLM-exposed Tagln^Cre^ mice (Figures 6A,B). Determination of RVSP in these mice revealed increased pressures in Tagln^Cre^ mice exposed to BLM compared to PBS groups, which is consistent with our previous studies (Karmouty-Quintana et al., 2012, 2015). In line with our morphometric data, BLM-treated Adora2b^f/f^-Tagln^Cre^ mice showed reduced RVSP levels compared to BLM-treated Tagln^Cre^ mice (Figure 6C). Determination of RVH using the Fulton index revealed evidence of RVH in BLM-treated Tagln^Cre^ mice that was attenuated in BLM-exposed Adora2b^f/f^-Tagln^Cre^ mice (Figure 6D). Consistent with previous publications (Sun et al., 2006; Zhou et al., 2010; Karmouty-Quintana et al., 2015), exposure to BLM led to increased levels of adenosine in BLM-treated Tagln^Cre^ mice that remained elevated in BLM-exposed Adora2b^f/f^-Tagln^Cre^ mice (Figure 6E). Collectively, these results demonstrate that conditional deletion of Adora2b using the Tagln^Cre^ promoter is able to attenuate markers of PH following BLM exposure.

### Vascular deletion of Adora2B does not alter fibrotic deposition following bleomycin (BLM)-induced lung injury

**Figure 7:**
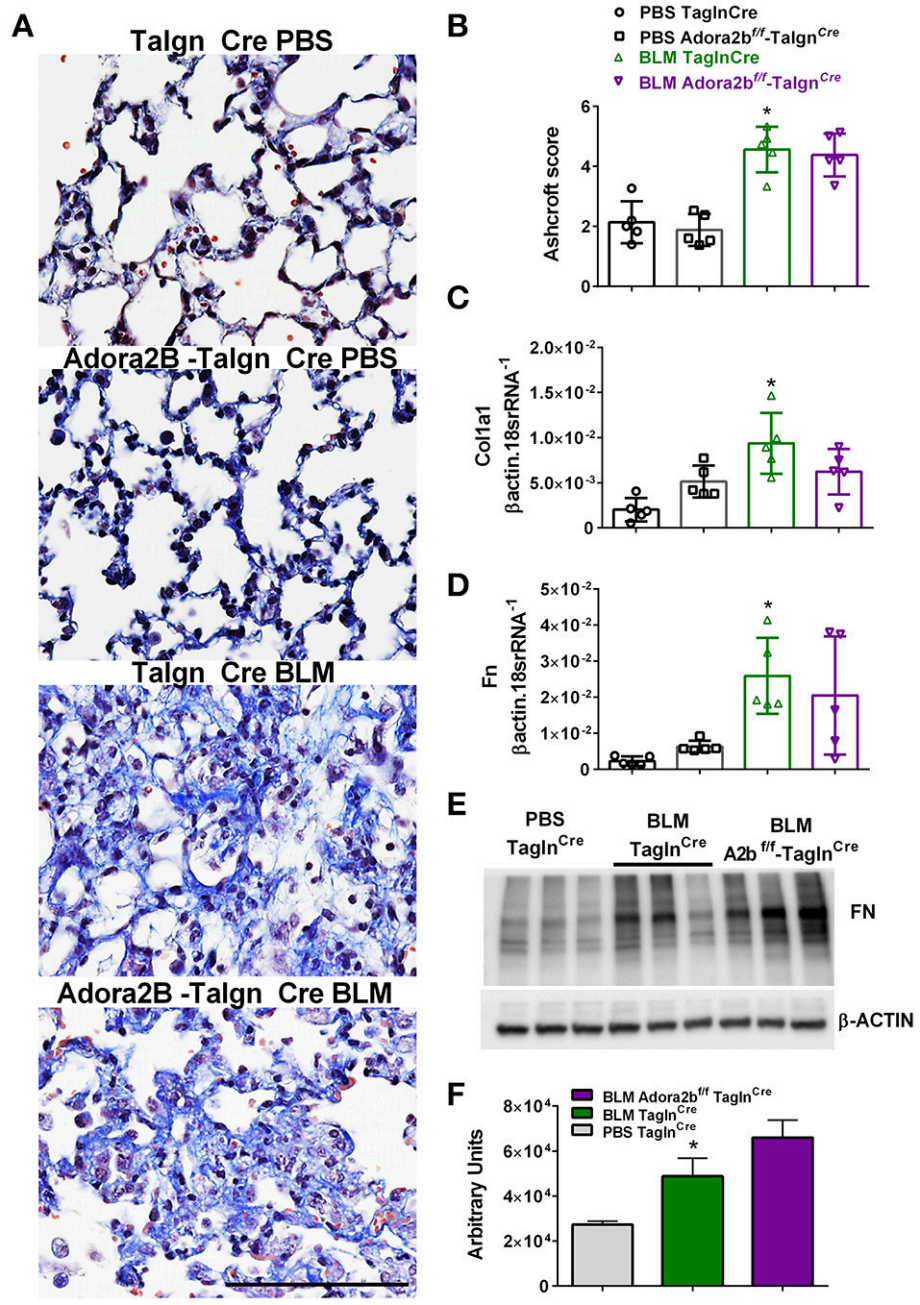
Fibrotic injury in Tagln^Cre^ and Adora2b^f/f−^Tagln^Cre^ mice exposed to bleomycin (BLM) or phosphate-buffered saline vehicle (PBS). All analyses were performed on day 33 of BLM and PBS exposure. **(A)** Representative Masson's Trichrome stained images showing fibrotic deposition in the parenchyma of PBS or BLM exposed Tagln^Cre^ or Adora2b^f/f−^Tagln^Cre^ mice and corresponding Ashcroft scores **(B)**. **(C)** Expression levels of Collagen 1a1 (Col1a1 **C**) Fibronectin (FN, **D**) by RT-PCR from PBS-Tagln^Cre^ mice (black circles and bar outline); PBS- Adora2b^f/f−^Tagln^Cre^ mice (black squares and bar outline); BLM-Tagln^Cre^ mice (green triangles and bar outline); BLM-Adora2b^f/f−^Tagln^Cre^ mice (magenta triangles and bar outline). Significant values: ^*^P < 0.05 refer to comparisons between Tagln^Cre^ BLM and Tagln^Cre^ PBS treatment groups. **(E)** Western blot for FN and β-actin for Tagln^Cre^ mice exposed to PBS or BLM and BLM-treated Adora2b^f/f−^Tagln^Cre^ mice. Densitometry analyses from Western Blot **(F)**. *N* = 5 for all groups for RT-PCR data an *N* = 3 for Western Blots.

Most remarkably, Adora2b^f/f^-Tagln^Cre^ mice exposed to BLM did not appear to show a reduction in fibrotic deposition as observed histologically in Masson's Trichome (MT) stained sections (Figure 7A) and in Ashcroft scores (Figure 7B). Expression analysis for collagen 1a2 (Col1a2) also reveal increased levels in the BLM- Tagln^Cre^ group that were not significantly reduced in the BLM-exposed Adora2b^f/f^-Tagln^Cre^ mice (Figure 7C). These observations are further supported by transcript levels for fibronectin (Fn), showing increased levels in BLM-exposed Tagln^Cre^ mice that are maintained in Adora2b^f/f^-Tagln^Cre^ mice exposed to BLM (Figure 7D). In line with these results, Western blots for Fn revealed increased signals for BLM-Tagln^Cre^ compared to PBS-Tagln^Cre^ mice that were maintained in the BLM-Adora2b^f/f^-Tagln^Cre^ group (Figures 7E,F). These results indicate that Adora2b-expressing Tagln^Cre^ cells plays a pivotal role in vascular remodeling in PH, but do not appear to modulate fibrotic responses in the lung, following BLM-exposure.

### Activation of ADORA2b in human pulmonary artery smooth muscle cells (PASMCs) leads to increased pro-remodeling mediators

**Figure 8:**
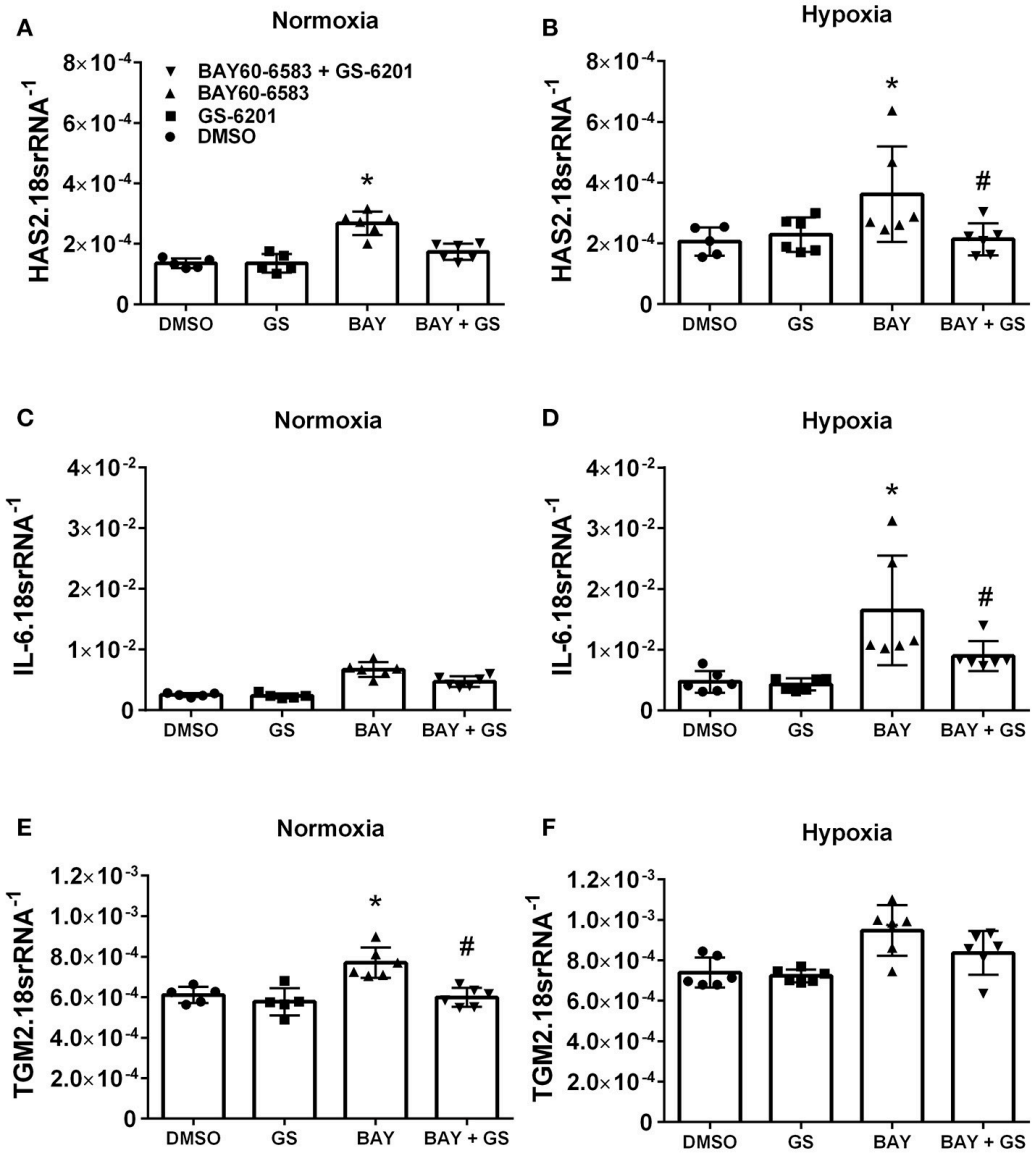
Response of primary human pulmonary artery smooth muscle cells (PASMCs) to activation of ADORA2B). Expression levels of HAS2 **(A,B)**; IL-6 **(C,D);** and TGM2 **(E,F)** under normoxia **(A–C)** or under hypoxia (2%O_2_, **D–F**) of human PASMC exposed to DMSO (white bars); the ADORA2B antagonist: GS-6201 (light gray bars); the ADORA2B agonist: BAY60-6583 (black bars) or under the presence of both GS-6201 and BAY60-6583 (dark gray bars). ^*^P < 0.05 refers to comparisons between DMSO and BAY60-6583 treatment groups and ^#^*P* < 0.05, are for comparisons between BAY60-6583 and BAY60-6583 + GS-6201 treatment groups. *N* = 5 for all groups.

Interestingly, our results demonstrate that deletion of Adora2b from smooth muscle cells is able to prevent the development of pulmonary hypertension in two distinct experimental models of PH. Thus, in order to evaluate the ADORA2B-mediated mechanisms that lead to PH, we performed experiments with isolated primary pulmonary artery smooth muscle cells (PASMCs) from normal healthy donors. In these experiments we report that activation of ADORA2B by the selective agonist BAY 60-6583 leads to increased expression levels of hyaluronan synthase 2 (HAS2) under both normoxia and hypoxia (Figures 8A,B); interleukin (IL)-6 only under hypoxia but not in normoxia (Figures 8C,D) and transglutaminase 2 (TGM2) under normoxia but not hypoxia (Figures 8E,F). It is interesting to note that the responses for HAS2 and IL-6 but not TGM2 were augmented under hypoxic conditions (Figures 7A–F). Remarkably, BAY60-6583-induced increased HAS2 and IL-6 signals were attenuated following treatment with GS-6201, a selective ADORA2B antagonist, in conditions of hypoxia, but not normoxia (Figures 8A–D). GS-6201 was only able to inhibit BAY 60-6583-induced up-regulation of TGM2 under normoxia but not in hypoxia (Figures 8E,F). Taken together, these results demonstrate an ADORA2B-dependent up-regulation of the pro-remodeling mediators HAS2, IL6, and TGM2, and that this response is further augmented under hypoxic conditions.

### Hyaluronan synthase 2 (Has2), IL-6 and transglutaminase 2 (Tgm2) levels in PH

**Figure 9:**
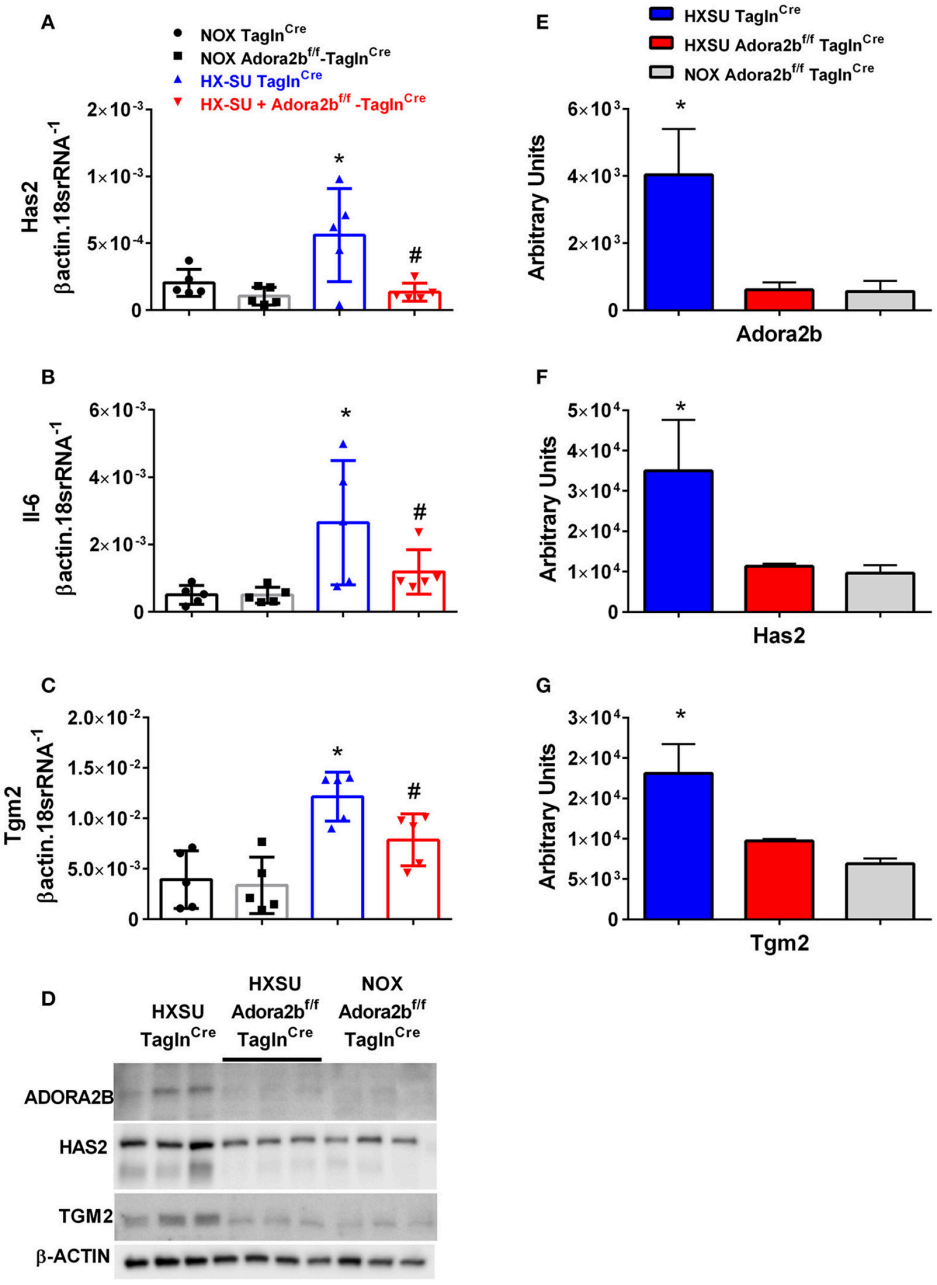
Expression levels of hyaluronan synthase 2 (Has2), interleukin-6 (Il-6) and transglutaminase 2 (Tgm2) in Tagln^Cre^ and Adora2b^f/f−^Tagln^Cre^ mice exposed to hypoxia-SUGEN (HX-SU) or normoxia (NOX). All analyses were performed on day 28 of HX-SU and NOX exposure. Has2 **(A)**; Il-6 **(B)**; Tgm2 **(C)**; expression levels measured by RT-PCR and normalized to the Geo mean of expression levels of βactin and 18srRNA from NOX-Tagln^Cre^ mice (black circles and bar outline); NOX- Adora2B^f/f−^Tagln^Cre^ mice (black squares and gray bar outline); HX-SU-Tagln^Cre^ mice (blue triangles and bar outline); HX-SU-Adora2B^f/f−^Tagln^Cre^ mice (red triangles and bar outline). Significant values: ^*^P < 0.05 refer to comparisons between Tagln^Cre^ HX-SU and Tagln^Cre^ NOX treatment groups. ^#^*P* < 0.05, are for comparisons between Tagln^Cre^ HX-SU and Adora2b^f/f−^Tagln^Cre^ + HX-SU treatment groups. **(D)** Western blot for Adora2b, Has2, Tgm2, and β-actin for Tagln^Cre^ mice exposed to HX-SU, HX-SU exposed Adora2b^f/f−^Tagln^Cre^ mice and NOX-treated Adora2b^f/f−^Tagln^Cre^ mice. Densitometry data for Adora2b **(E)**, Has2 **(F)** and Tgm2 **(G)**: ^*^P < 0.05 refer to comparisons between Adora2b-Tagln^Cre^ HX-SU and Tagln^Cre^ HX-SU treatment groups. *N* = 5 for all RT-PCR experiments and *N* = 3 for western blots.

We next examined whether expression levels of hyaluronan synthase 2 (Has2), Il-6 and transglutaminase 2 (Tgm2) were altered in mice exposed to HX-SU. Consistent with our data, HX-SU-Tagln^Cre^ mice presented with higher levels of Has2, IL6, and Tgm2 that were attenuated in HX-SU-Adora2b^f/f^-Tagln^Cre^ mice (Figures 9A–C). These observations are consistent with Western blots from mice showing reduced expression levels of ADORA2B, HAS2, and TGM2 in HX-SU-Adora2b^f/f^-Tagln^Cre^ and NOX-Adora2b^f/f^-Tagln^Cre^ mice compared to Adora2b-competent-HX-SU-exposed Tagln^Cre^ mice (Figure 9D) and subsequent densitometry analyses (Figures 9E–G).

**Figure 10:**
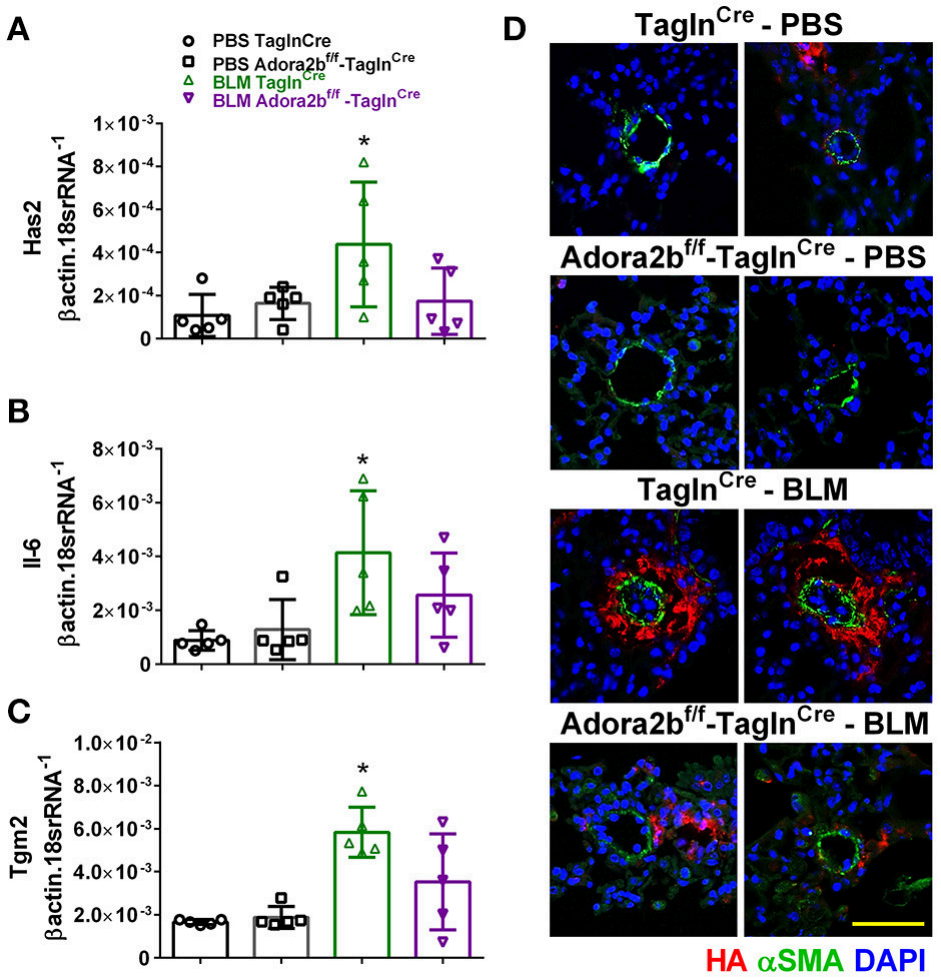
Expression levels of hyaluronan synthase 2 (HAS2), interleukin-6 (IL-6) and transglutaminase 2 (Tgm2) in TaglnCre and Adora2b^f/f−^Tagln^Cre^ mice exposed to bleomycin (BLM) or vehicle phosphate-buffered saline (PBS). Has2 **(A)**; IL-6 **(B)**; Tgm2 **(C)**; from PBS-Tagln^Cre^ mice (black circles and bar outline); PBS- Adora2b^f/f−^Tagln^Cre^ mice (black squares and bar outline); BLM-Tagln^Cre^ mice (green triangles and bar outline); BLM-Adora2b^f/f−^Tagln^Cre^ mice (magenta triangles and bar outline). Significant values: ^*^P < 0.05 refer to comparisons between TaglnCre BLM and TaglnCre PBS treatment groups. **(D)** Immunohistochemistry from representative vessels from PBS or BLM exposed Tagln^Cre^ and Adora2b^f/f−^Tagln^Cre^ mice stained for alpha smooth muscle actin (αSMA, green signals), hyaluronan (red signals) and counter stained with DAPI (blue signals). Scale bar represents 50 μm. *N* = 5 for all groups.

**Figure 11:**
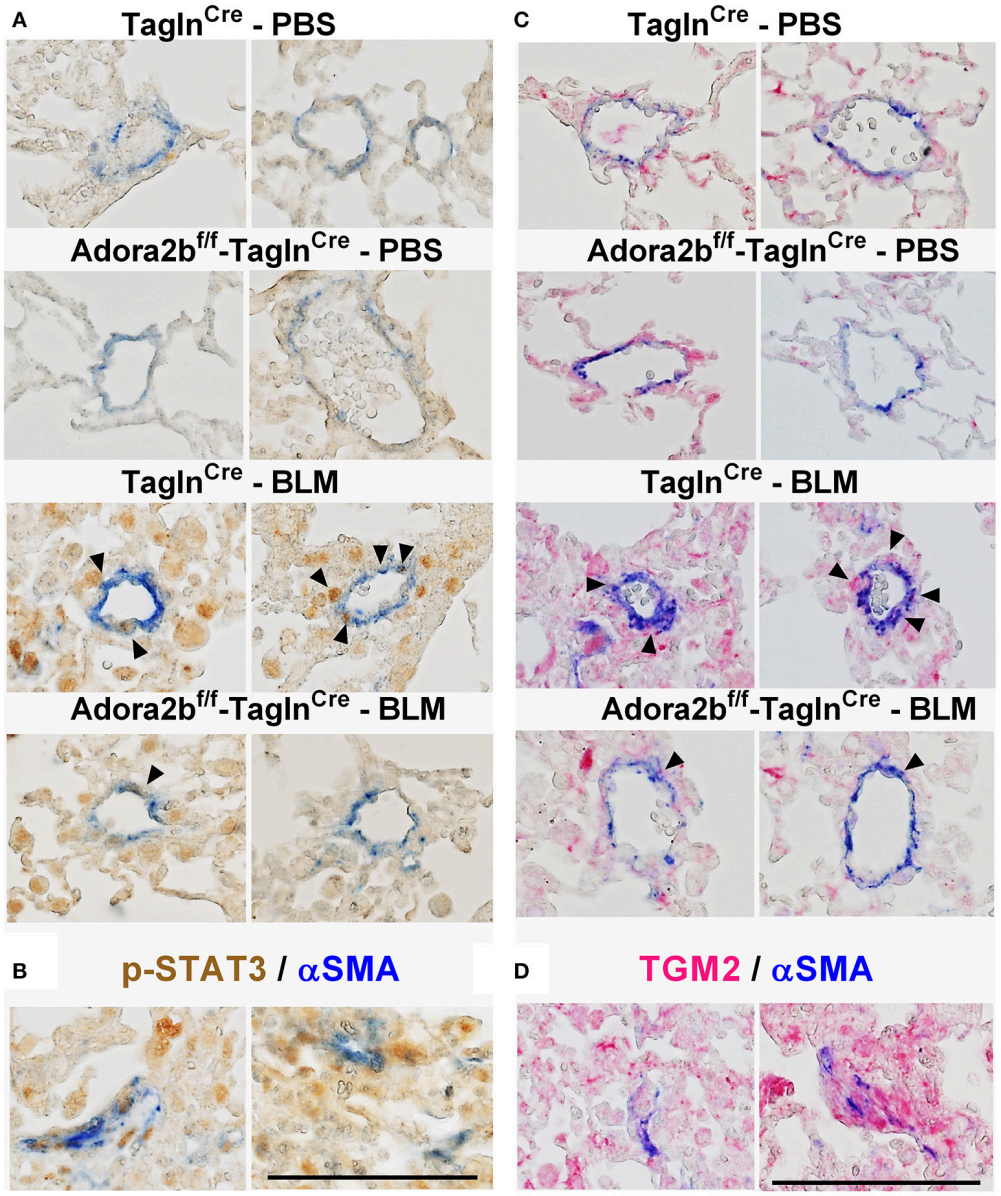
Dual immunohistochemistry (IHC) from representative vessels from PBS or BLM exposed Tagln^Cre^ and Adora2b^f/f−^Tagln^Cre^ mice stained for **(A)** alpha smooth muscle actin (αSMA, blue) and nuclear P-STAT3 (brown signals); **(B)** represent fibrotic areas rich in myofibroblasts (αSMA positive) where PSTAT is present; **(C)** αSMA, blue and transglutaminase (TGM2, red signals) **(D)** represents fibrotic areas rich in myofibroblasts (αSMA positive) where TGM2 is also present. Scale bar represents 200 μm. Arrow heads point and p-STAT3 or TGM2 positive cells in the vessel wall (αSMA positive).

**Figure 12:**
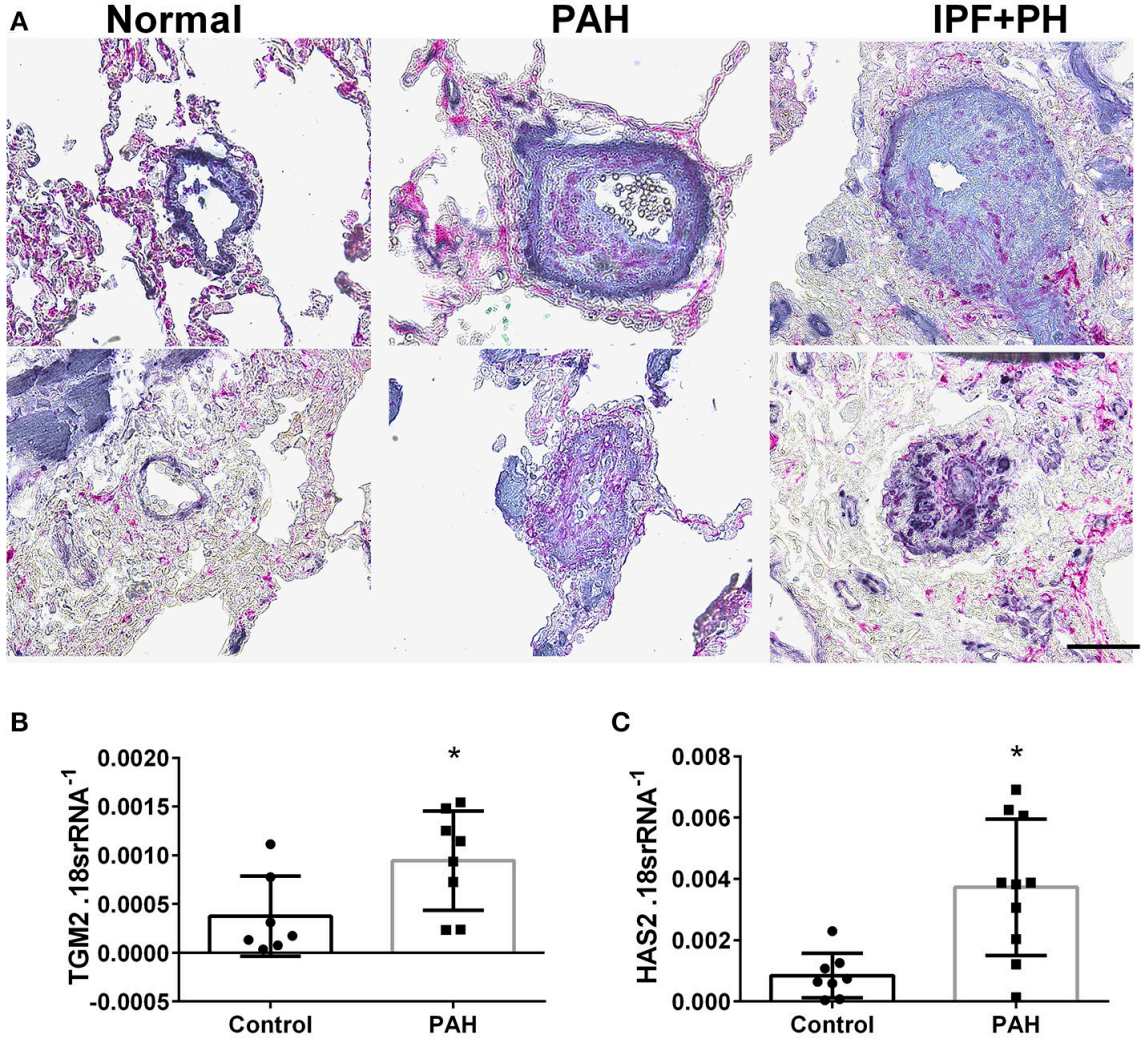
**(A)** Dual immunohistochemistry (IHC) for alpha smooth muscle actin (αSMA, indigo-blue signals) and transglutaminase (TGM2, pink-magenta signals) from representative pulmonary arteries from normal (top), pulmonary arterial hypertension (PAH, middle), or IPF+PH (lower) lungs. Scale bar represents 200 μm. Expression levels of TGM2 **(B)** and HAS2 **(C)** from PASMCs isolated from controls and patients with iPAH. ^*^P < 0.05 refer to comparisons between Control and PAH groups. *N* = 8 for all groups.

In our experimental model of BLM-induced lung fibrosis and PH, we report increased levels of Has2, Il-6 and Tgm2 following BLM exposure compared to PBS exposure in Tagln^Cre^ mice (Figures 10A–C). No significant difference in Has2, Il-6 or Tgm2 transcript levels were observed between BLM exposed Adora2b^f/f^-Talgn^Cre^ mice compared to the BLM Talgn^Cre^ group (Figures 10A–C). However, vascular hyaluronan deposition was reduced in BLM-exposed Adora2b^f/f^-Talgn^Cre^ mice compared to BLM-exposed Talgn^Cre^ mice (Figure 10D). Our RT-PCR findings were consistent with increased signals from Western blots for P-STAT3 (but not STAT3) and HAS2 for BLM-exposed Talgn ^Cre^ mice compared to PBS exposed Talgn ^Cre^ mice that remained elevated in BLM exposed Adora2b^f/f^-Talgn^Cre^ mice, albeit no differences in TGM2 were identified (Supplementary Figures 3A–E). IHC staining for P-STAT3 and TGM2 revealed increased signals in the vasculature of BLM-exposed Talgn^Cre^ mice compared to PBS-treated mice that were attenuated in BLM-exposed Adora2b^f/f^-Talgn^Cre^ mice (Figures 11A,C). Consistent with the presence of fibrosis in these mice, we report increased signals for P-STAT3 and TGM2 in fibrotic areas rich in myofibroblasts (Figures 11B,D). In addition to augmented TGM2 levels in HX-SU and BLM-exposed mice, we report increased TGM2 signals within remodeled vessels in patients with a diagnosis of PAH or IPF + PH (Figure 12A). These observations were in line increased TGM2 transcripts from PAH patients compared to controls (Figure 12B) and with increased HAS2 mRNA in PAH vs. control PASMCs (Figure 12C). Taken together, our results show a likely mechanism where Adora2b activation promotes the development of PH through increased expression of Has2, Il6 and Tgm2 in experimental models of disease and in human patients with Group I or Group III PH.

## Discussion

The presence of pulmonary hypertension (PH) in the context of idiopathic pulmonary fibrosis (IPF) is associated with significantly increased morbidity and mortality rates (Behr and Ryu, 2008; Pitsiou et al., 2011; Collum et al., 2017a). Despite the detrimental consequences of PH, there are very limited therapies available that can treat PH in IPF patients (Collum et al., 2017a). Although anti-fibrotic agents have been shown to attenuate experimental fibrosis and PH (Hemnes et al., 2008; Schroll et al., 2010, 2013; Van Rheen et al., 2011; Karmouty-Quintana et al., 2012, 2015; Bei et al., 2013; Grasemann et al., 2015; Chen et al., 2016; Avouac et al., 2017), these therapies have not translated to the clinic. Moreover, standard therapies for PAH have failed to show a benefit in Group III PH and have been associated with worsening mortality (Ruggiero et al., 2012; Klinger, 2016).

A major finding of our study is that switching-off Adora2b in vascular smooth muscle cells using the Tagln promoter is able to prevent the development of PH in two distinct mouse models: the HX-SU and the BLM model. Most remarkably, we report that Adora2b^f/f^-Tagln^Cre^ mice exposed to BLM still present with fibrotic lung injury that is not significantly different to that of control Tagln^Cre^ mice exposed to BLM. These results demonstrate that Adora2b expression in the vascular smooth muscle is a viable target to treat PH, either on its own or associated with chronic lung injury. Consistent with previous publications from our group, we demonstrate that the likely mechanism for Adora2b-mediated PH is through enhanced IL-6 and hyaluronic acid deposition (Karmouty-Quintana et al., 2012, 2013a, 2015; Collum et al., 2017b). In addition to these meditators, we also report the novel finding that Tgm2 can be modulated by Adora2b and that this is consistent with increased expression of vascular Tgm2 in experimental models of PH and in remodeled vessels of patients with a diagnosis of PAH or IPF+PH.

In response to injury or stress, the lung releases ATP that is then converted to adenosine by ecto-nucleotidases (Colgan et al., 2006). This response is part of a normal physiological reaction to high altitude (Song et al., 2017; Sun et al., 2017) and to acute lung injury (Van Linden and Eltzschig, 2007; Eckle et al., 2009). These ecto-nucleotidases include CD39 and CD73 that are regulated by hypoxia-inducible factor (HIF-1A) (Synnestvedt et al., 2002; Hart et al., 2010). Adenosine is then able to activate one of its four G-protein coupled receptors: the adenosine A1 (ADORA1), A2A (ADORA2A), A2B (ADORA2B), and A3 (ADORA3) receptors (Fredholm et al., 2001). All four of these receptors have been shown to be involved in cellular processes implicated in tissue injury (Karmouty-Quintana et al., 2013b). Under normal physiological conditions, adenosine is rapidly metabolized to inosine by adenosine deaminase (ADA) (Fredholm et al., 2001) or up-taken intracellularly through equilibrative nucleotide transporters (ENTs) (Eltzschig et al., 2005). Studies in CD73 and CD39 deficient mice have demonstrated that increased adenosine levels are critical for the resolution of acute lung injury (Volmer et al., 2006; Eckle et al., 2007; Davies et al., 2014). Yet, despite the protective effects of adenosine, sustained levels have been shown to mediate chronic lung injury (Blackburn, 2003; Blackburn and Kellems, 2005; Chunn et al., 2006). These studies are consistent with increased expression of CD73 and ADORA2B in experimental models and patients with lung injury (Chunn et al., 2006; Zhou et al., 2010; Wirsdörfer et al., 2016). Lowering extracellular adenosine through treatment with ADA or genetic deletion of CD73 have shown that these mice are protected from the development of chronic lung injury (Chunn et al., 2006; Zhou et al., 2010; Wirsdörfer et al., 2016). This dualistic action of adenosine can be explained by the concept of purinergic remodeling, where alterations in the expression levels of enzymes of adenosine metabolism (CD73, ADA), transport proteins such as ENTs, or adenosine receptors may influence the progression from acute to chronic responses (Zhou et al., 2009). A highly relevant example of purinergic remodeling is the altered levels of CD39, CD73, ENTs and adenosine receptors in IPF+PH (Garcia-Morales et al., 2016). Here, purinergic remodeling shifted the balance toward accumulation of adenosine and increased expression of ADORA2B (Garcia-Morales et al., 2016). Interestingly, in our study, although adenosine levels were maintained, vascular remodeling and RVSP were attenuated in both the HX-SU and the BLM model. Yet these increased levels of adenosine were likely responsible for the presence of RVH following HX-SU exposure (Toldo et al., 2012; Ferrari et al., 2016) and for the development of fibrosis (Blackburn and Kellems, 2005; Chunn et al., 2006; Sun et al., 2006).

Intriguingly, in the context of PH, reduced adenosine levels in the pulmonary circulation were reported in patients with COPD + PH and in Primary Pulmonary Hypertension (PPH) compared to normal individuals (Saadjian et al., 1999). Although the sample size is limited (*n* = 7 controls, *n* = 8 COPD+PH, and *n* = 8 PPH) these results suggest that reduced adenosine levels in the circulation are pathogenic (Saadjian et al., 1999). At face-value these observations are counterintuitive to our hypothesis that enhanced ADORA2B levels are pathogenic in PH since ADORA2B has the lowest affinity for adenosine (Fredholm et al., 2001). Despite this, it is important to consider that adenosine has a very low half-life and is rapidly metabolized *in vivo* and *in vitro* (Klabunde, 1983). Thus it is conceivable that local elevations of tissue adenosine near the vasculature lead to enhanced activation of vascular smooth cell ADORA2B and promote the development of PH. In line with these studies, we have reported increased activity of CD73 and reduced activity of ADA in tissue samples of patients with IPF+PH (Garcia-Morales et al., 2016) that point to a pathogenic accumulation of adenosine in PH. Similarly, we show that PASMCs from patients with PAH present with increased CD73 that point at an enhanced capacity to accumulate adenosine in remodeled vessels in PAH.

Augmented ADORA2B levels have been reported in patients with IPF and COPD (Zhou et al., 2010; Karmouty-Quintana et al., 2013b) that are consistent with increased Adora2b levels in experimental models of chronic lung injury that also present with PH (Sun et al., 2006; Pedroza et al., 2011; Karmouty-Quintana et al., 2012, 2013a). Using an Adora2b antagonist and *global* Adora2b-deficient mice, we have shown that abrogation of this receptor reduces PH associated with chronic lung disease (Karmouty-Quintana et al., 2012, 2013a). In these experiments, the attenuation of PH was linked with reduced severity of lung injury (Karmouty-Quintana et al., 2012, 2013a). Similarly, conditional deletion of myeloid ADORA2B resulted in reduced fibro-proliferative lesions and attenuated markers of PH following exposure to BLM (Karmouty-Quintana et al., 2015). Taken together, these results demonstrated the therapeutic potential of abrogation of ADORA2B signaling in lung injury and PH; however, it was not known whether vascular ADORA2B could contribute directly to the development of PH in an *in vivo* setting. Similarly, the role of ADORA2B in clinical or experimental models of PAH had not been addressed until now (Alencar et al., 2017). Strikingly, our results show that switching-off vascular smooth muscle ADORA2B is able to prevent HX-SU and BLM induced PH without altering fibrotic deposition levels in BLM-exposed mice. These results provide further rationale for the use of ADORA2B antagonists for the treatment of PAH and PH associated with chronic lung injury. We believe that this discrepancy is the result of the pleiotropic functions of ADORA2B; where ADORA2B activation in other cells such as fibroblasts and myeloid cells may continue to drive the fibrotic process (Sun et al., 2006; Karmouty-Quintana et al., 2013b, 2015). Further studies testing the effect of ADORA2B antagonists in established models replicating features of PAH would provide important pre-clinical data supporting the use of ADORA2B antagonists for the treatment of this fatal condition.

Regarding the ADORA2B mediated mechanisms, our data showed that activation of ADORA2B in PASMCs leads to increased levels of IL-6 and hyaluronan, two mediators that have been associated in the pathophysiology of lung fibrosis and PH (Karmouty-Quintana et al., 2012, 2015; Chen et al., 2016; Collum et al., 2017b). Indeed increased IL-6 has also been shown to play an important role in the development of PAH (Ricard et al., 2014). In fact, treatment with a hyaluronan synthase inhibitor attenuates BLM-induced PH independent from fibrotic deposition in the BLM model of lung injury (Collum et al., 2017b). Interestingly, we also show that activation of Adora2b modulates expression of Tgm2, a multifunctional enzyme (Gundemir et al., 2012; Eckert et al., 2014; Liu et al., 2017) that has been associated with PH (Diraimondo et al., 2014; Penumatsa et al., 2017) and pulmonary fibrosis (Oh et al., 2011; Olsen et al., 2011, 2014). Tgm2 likely contributes to the development of PH through serotonylation leading to enhanced cellular proliferation (Diraimondo et al., 2014; Penumatsa et al., 2014, 2017). However, Tgm2 can also crosslink with other ECM proteins that contribute to vascular stiffening (Gundemir et al., 2012; Eckert et al., 2014) similar to vascular expression of hyaluronan (Karmouty-Quintana et al., 2013a). Tgm2 could also promote pulmonary hypertension by other means (Liu et al., 2017) that may affect receptor levels by altering ubiquitination or influence the presence of auto-antibodies (Liu et al., 2014, 2015).

It is also important to consider that other adenosine receptors may a play a role in PH. An elegant review (Alencar et al., 2017) has recently summed up the most recent developments of adenosine receptors for the treatment of PAH. Most notably activation of Adora2a has been shown to be effective for the treatment of PAH (Alencar et al., 2017); this is consistent with our data showing reduced protein levels of Adora2a in PASMCs isolated from patients with iPAH.

In conclusion, our data show that conditional deletion of Adora2b from vascular smooth muscle cells prevents the development of PH through a mechanism that involves upregulation of IL-6, hyaluronan and Tgm2. Taken together, these results point at a role for inhibition of Adora2b for the treatment of Group I and Group III PH.

## Ethics statement

This study was carried out in accordance with the recommendations of the UTHealth Animal Welfare Committee (AWC), an AAALAC accredited Institution. The protocol was approved by the AWC under protocol number AWC-16-00060. The use of human derived material was reviewed by institutional review board (IRB): HSC-MS-08-0354.

## Author contributions

TM, AH, and HK-Q planned and performed experiments, acquired and analyzed data and wrote the manuscript. LT, CP, SC, N-YC, TW, and CL performed experiments, acquired and analyzed data. HE provided the floxed Adora2b mice and together with JD and YX contributed to the design of the study and interpretation of the results. SJ, KR, AG, and BB supervised the use of human samples for the study. MB, CG, and HK-Q conceived the idea, designed and supervised the study. All authors discussed the results and contributed to the final manuscript.

### Conflict of interest statement

The authors declare that the research was conducted in the absence of any commercial or financial relationships that could be construed as a potential conflict of interest.

## References

[B1] Alencar A. K. N. Montes G. C. Barreiro E. J. Sudo R. T. Zapata-Sudo G. (2017). Adenosine receptors as drug targets for treatment of pulmonary arterial hypertension. Front. Pharmacol. 8:858. 10.3389/fphar.2017.00858 29255415 PMC5722832

[B2] Archer S. L. Weir E. K. Wilkins M. R. (2010). Basic science of pulmonary arterial hypertension for clinicians: new concepts and experimental therapies. Circulation 121, 2045–2066. 10.1161/CIRCULATIONAHA.108.847707 20458021 PMC2869481

[B3] Avouac J. Konstantinova I. Guignabert C. Pezet S. Sadoine J. Guilbert T.. (2017). Pan-PPAR agonist IVA337 is effective in experimental lung fibrosis and pulmonary hypertension. Ann. Rheum. Dis. 76, 1931–1940. 10.1136/annrheumdis-2016-210821 28801346

[B4] Behr J. Ryu J. H. (2008). Pulmonary hypertension in interstitial lung disease. Eur. Respir. J. 31, 1357–1367. 10.1183/09031936.00171307 18515559

[B5] Bei Y. Hua-Huy T. Duong-Quy S. Nguyen V. H. Chen W. Nicco C.. (2013). Long-term treatment with fasudil improves bleomycin-induced pulmonary fibrosis and pulmonary hypertension via inhibition of Smad2/3 phosphorylation. Pulm. Pharmacol. Ther. 26, 635–643. 10.1016/j.pupt.2013.07.008 23928001

[B6] Blackburn M. R. (2003). Too much of a good thing: adenosine overload in adenosine-deaminase-deficient mice. Trends Pharmacol. Sci. 24, 66–70. 10.1016/S0165-6147(02)00045-7 12559769

[B7] Blackburn M. R. Kellems R. E. (2005). Adenosine deaminase deficiency: metabolic basis of immune deficiency and pulmonary inflammation. Adv. Immunol. 86, 1–41. 10.1016/S0065-2776(04)86001-2 15705418

[B8] Chen N. Y. D Collum S. Luo F. Weng T. Le T. T. M Hernandez A.. (2016). Macrophage bone morphogenic protein receptor 2 depletion in idiopathic pulmonary fibrosis and Group III pulmonary hypertension. Am. J. Physiol. Lung Cell. Mol. Physiol. 311, L238–254. 10.1152/ajplung.00142.2016 27317687 PMC6425517

[B9] Chunn J. L. Mohsenin A. Young H. W. Lee C. G. Elias J. A. Kellems R. E.. (2006). Partially adenosine deaminase-deficient mice develop pulmonary fibrosis in association with adenosine elevations. Am. J. Physiol. Lung Cell. Mol. Physiol. 290, L579–L587. 10.1152/ajplung.00258.2005 16258000

[B10] Ciuclan L. Bonneau O. Hussey M. Duggan N. Holmes A. M. Good R.. (2011). A novel murine model of severe pulmonary arterial hypertension. Am. J. Respir. Crit. Care Med. 184, 1171–1182. 10.1164/rccm.201103-0412OC 21868504

[B11] Colgan S. P. Eltzschig H. K. Eckle T. Thompson L. F. (2006). Physiological roles for ecto-5′-nucleotidase (CD73). Purinergic Signal. 2, 351–360. 10.1007/s11302-005-5302-5 18404475 PMC2254482

[B12] Collum S. D. Amione-Guerra J. Cruz-Solbes A. S. DiFrancesco A. Hernandez A. M. Hanmandlu A. (2017a). Pulmonary hypertension associated with idiopathic pulmonary fibrosis: current and future perspectives. Can. Respir. J. 2017:1430350 10.1155/2017/1430350 28286407 PMC5327768

[B13] Collum S. D. Chen N. Y. Hernandez A. M. Hanmandlu A. Sweeney H. Mertens T. C. J.. (2017b). Inhibition of hyaluronan synthesis attenuates pulmonary hypertension associated with lung fibrosis. Br. J. Pharmacol. 174, 3284–3301. 10.1111/bph.13947 28688167 PMC5595757

[B14] Davies J. Karmouty-Quintana H. Le T. T. Chen N. Y. Weng T. Luo F.. (2014). Adenosine promotes vascular barrier function in hyperoxic lung injury. Physiol Rep 2:e12155. 10.14814/phy2.12155 25263205 PMC4270235

[B15] Diraimondo T. R. Klöck C. Warburton R. Herrera Z. Penumatsa K. Toksoz D.. (2014). Elevated transglutaminase 2 activity is associated with hypoxia-induced experimental pulmonary hypertension in mice. ACS Chem. Biol. 9, 266–275. 10.1021/cb4006408 24152195 PMC3947056

[B16] Eckert R. L. Kaartinen M. T. Nurminskaya M. Belkin A. M. Colak G. Johnson G. V.. (2014). Transglutaminase regulation of cell function. Physiol. Rev. 94, 383–417. 10.1152/physrev.00019.2013 24692352 PMC4044299

[B17] Eckle T. Füllbier L. Wehrmann M. Khoury J. Mittelbronn M. Ibla J.. (2007). Identification of ectonucleotidases CD39 and CD73 in innate protection during acute lung injury. J. Immunol. 178, 8127–8137. 10.4049/jimmunol.178.12.8127 17548651

[B18] Eckle T. Koeppen M. Eltzschig H. K. (2009). Role of extracellular adenosine in acute lung injury. Physiology 24, 298–306. 10.1152/physiol.00022.2009 19815856

[B19] Eltzschig H. K. Abdulla P. Hoffman E. Hamilton K. E. Daniels D. Schönfeld C.. (2005). HIF-1-dependent repression of equilibrative nucleoside transporter (ENT) in hypoxia. J. Exp. Med. 202, 1493–1505. 10.1084/jem.20050177 16330813 PMC2213326

[B20] Farkas L. Gauldie J. Voelkel N. F. Kolb M. (2011). Pulmonary hypertension and idiopathic pulmonary fibrosis: a tale of angiogenesis, apoptosis, and growth factors. Am. J. Respir. Cell Mol. Biol. 45, 1–15. 10.1165/rcmb.2010-0365TR 21057104

[B21] Fell C. D. (2012). Idiopathic pulmonary fibrosis: phenotypes and comorbidities. Clin. Chest Med. 33, 51–57. 10.1016/j.ccm.2011.12.005 22365245

[B22] Ferrari D. Gambari R. Idzko M. Müller T. Albanesi C. Pastore S.. (2016). Purinergic signaling in scarring. FASEB J. 30, 3–12. 10.1096/fj.15-274563 26333425 PMC4684510

[B23] Fredholm B. B. (2007). Adenosine, an endogenous distress signal, modulates tissue damage and repair. Cell Death Differ. 14, 1315–1323. 10.1038/sj.cdd.4402132 17396131

[B24] Fredholm B. B. IJzerman A.P. Jacobson K. A. Klotz K. N. Linden J. (2001). International Union of Pharmacology. XXV. Nomenclature and classification of adenosine receptors. Pharmacol. Rev. 53, 527–552. 10.1016/j.neuropharm.2015.12.001 11734617 PMC9389454

[B25] Garcia-Morales L. J. Chen N. Y. Weng T. Luo F. Davies J. Philip K.. (2016). Altered hypoxic-adenosine axis and metabolism in group III pulmonary hypertension. Am. J. Respir. Cell Mol. Biol. 54, 574–583. 10.1165/rcmb.2015-0145OC 26414702 PMC4821053

[B26] Grasemann H. Dhaliwal R. Ivanovska J. Kantores C. Mcnamara P. J. Scott J. A.. (2015). Arginase inhibition prevents bleomycin-induced pulmonary hypertension, vascular remodeling, and collagen deposition in neonatal rat lungs. Am. J. Physiol. Lung Cell. Mol. Physiol. 308, L503–L510. 10.1152/ajplung.00328.2014 25595650

[B27] Guignabert C. Raffestin B. Benferhat R. Raoul W. Zadigue P. Rideau D.. (2005). Serotonin transporter inhibition prevents and reverses monocrotaline-induced pulmonary hypertension in rats. Circulation 111, 2812–2819. 10.1161/CIRCULATIONAHA.104.524926 15927991

[B28] Gundemir S. Colak G. Tucholski J. Johnson G. V. (2012). Transglutaminase 2: a molecular Swiss army knife. Biochim. Biophys. Acta 1823, 406–419. 10.1016/j.bbamcr.2011.09.012 22015769 PMC3265640

[B29] Hansdottir S. Groskreutz D. J. Gehlbach B. K. (2013). WHO's in second?: A practical review of World Health Organization group 2 pulmonary hypertension. Chest 144, 638–650. 10.1378/chest.12-2114 23918108 PMC3734892

[B30] Hart M. L. Gorzolla I. C. Schittenhelm J. Robson S. C. Eltzschig H. K. (2010). SP1-dependent induction of CD39 facilitates hepatic ischemic preconditioning. J. Immunol. 184, 4017–4024. 10.4049/jimmunol.0901851 20207994 PMC2846294

[B31] Hemnes A. R. Zaiman A. Champion H. C. (2008). PDE5A inhibition attenuates bleomycin-induced pulmonary fibrosis and pulmonary hypertension through inhibition of ROS generation and RhoA/Rho kinase activation. Am. J. Physiol. Lung Cell. Mol. Physiol. 294, L24–L33. 10.1152/ajplung.00245.2007 17965319

[B32] Hübner R. H. Gitter W. El Mokhtari N. E. Mathiak M. Both M. Bolte H.. (2008). Standardized quantification of pulmonary fibrosis in histological samples. Biotechniques 44, 507–511, 514–507. 10.2144/000112729 18476815

[B33] Huertas A. Tu L. Thuillet R. Le Hiress M. Phan C. Ricard N.. (2015). Leptin signalling system as a target for pulmonary arterial hypertension therapy. Eur. Respir. J. 45, 1066–1080. 10.1183/09031936.00193014 25745038

[B34] Judge E. P. Fabre A. Adamali H. I. Egan J. J. (2012). Acute exacerbations and pulmonary hypertension in advanced idiopathic pulmonary fibrosis. Eur. Respir. J. 40, 93–100. 10.1183/09031936.00115511 22135282

[B35] Karmouty-Quintana H. Philip K. Acero L. F. Chen N. Y. Weng T. Molina J. G.. (2015). Deletion of ADORA2B from myeloid cells dampens lung fibrosis and pulmonary hypertension. FASEB J. 29, 50–60. 10.1096/fj.14-260182 25318478 PMC4763976

[B36] Karmouty-Quintana H. Weng T. Garcia-Morales L. J. Chen N. Y. Pedroza M. Zhong H. (2013a). ADORA2B and hyaluronan modulate pulmonary hypertension associated with chronic obstructive pulmonary disease. Am. J. Respir. Cell Mol. Biol. 49, 1038–1047. 10.1165/rcmb.2013-0089OC 23855769 PMC5459551

[B37] Karmouty-Quintana H. Xia Y. Blackburn M. R. (2013b). Adenosine signaling during acute and chronic disease states. J. Mol. Med. 91, 173–181. 10.1007/s00109-013-0997-1 23340998 PMC3606047

[B38] Karmouty-Quintana H. Zhong H. Acero L. Weng T. Melicoff E. West J. D.. (2012). The A2B adenosine receptor modulates pulmonary hypertension associated with interstitial lung disease. FASEB J. 26, 2546–2557. 10.1096/fj.11-200907 22415303 PMC3650483

[B39] Klabunde R. E. (1983). Dipyridamole inhibition of adenosine metabolism in human blood. Eur. J. Pharmacol. 93, 21–26. 10.1016/0014-2999(83)90026-2 6628545

[B40] Klinger J. R. (2016). Group III pulmonary hypertension: pulmonary hypertension associated with lung disease: epidemiology, pathophysiology, and treatments. Cardiol. Clin. 34, 413–433. 10.1016/j.ccl.2016.04.003 27443138

[B41] Lee K. J. Czech L. Waypa G. B. Farrow K. N. (2013). Isolation of pulmonary artery smooth muscle cells from neonatal mice. J. Vis. Exp. e50889. 10.3791/50889 24193306 PMC3943298

[B42] Lennon P. F. Taylor C. T. Stahl G. L. Colgan S. P. (1998). Neutrophil-derived 5'-adenosine monophosphate promotes endothelial barrier function via CD73-mediated conversion to adenosine and endothelial A2B receptor activation. J. Exp. Med. 188, 1433–1443. 10.1084/jem.188.8.1433 9782120 PMC2213403

[B43] Liu C. Kellems R. E. Xia Y. (2017). Inflammation, autoimmunity, and hypertension: the essential role of tissue transglutaminase. Am. J. Hypertens. 30, 756–764. 10.1093/ajh/hpx027 28338973 PMC5861548

[B44] Liu C. Luo R. Elliott S. E. Wang W. Parchim N. F. Iriyama T.. (2015). Elevated transglutaminase activity triggers angiotensin receptor activating autoantibody production and pathophysiology of Preeclampsia. J Am Heart Assoc 4:e002323. 10.1161/JAHA.115.002323 26675250 PMC4845265

[B45] Liu C. Wang W. Parchim N. Irani R. A. Blackwell S. C. Sibai B.. (2014). Tissue transglutaminase contributes to the pathogenesis of preeclampsia and stabilizes placental angiotensin receptor type 1 by ubiquitination-preventing isopeptide modification. Hypertension 63, 353–361. 10.1161/HYPERTENSIONAHA.113.02361 24191290 PMC4052572

[B46] Morrell N. W. Yang X. Upton P. D. Jourdan K. B. Morgan N. Sheares K. K.. (2001). Altered growth responses of pulmonary artery smooth muscle cells from patients with primary pulmonary hypertension to transforming growth factor-beta(1) and bone morphogenetic proteins. Circulation 104, 790–795. 10.1161/hc3201.094152 11502704

[B47] Oh K. Park H. B. Byoun O. J. Shin D. M. Jeong E. M. Kim Y. W.. (2011). Epithelial transglutaminase 2 is needed for T cell interleukin-17 production and subsequent pulmonary inflammation and fibrosis in bleomycin-treated mice. J. Exp. Med. 208, 1707–1719. 10.1084/jem.20101457 21746810 PMC3149214

[B48] Olsen K. C. Epa A. P. Kulkarni A. A. Kottmann R. M. Mccarthy C. E. Johnson G. V.. (2014). Inhibition of transglutaminase 2, a novel target for pulmonary fibrosis, by two small electrophilic molecules. Am. J. Respir. Cell Mol. Biol. 50, 737–747. 10.1165/rcmb.2013-0092OC 24175906 PMC4068920

[B49] Olsen K. C. Sapinoro R. E. Kottmann R. M. Kulkarni A. A. Iismaa S. E. Johnson G. V.. (2011). Transglutaminase 2 and its role in pulmonary fibrosis. Am. J. Respir. Crit. Care Med. 184, 699–707. 10.1164/rccm.201101-0013OC 21700912 PMC3208598

[B50] Pedroza M. Schneider D. J. Karmouty-Quintana H. Coote J. Shaw S. Corrigan R.. (2011). Interleukin-6 contributes to inflammation and remodeling in a model of adenosine mediated lung injury. PLoS ONE 6:e22667. 10.1371/journal.pone.0022667 21799929 PMC3143181

[B51] Penumatsa K. C. Toksoz D. Warburton R. R. Hilmer A. J. Liu T. Khosla C.. (2014). Role of hypoxia-induced transglutaminase 2 in pulmonary artery smooth muscle cell proliferation. Am. J. Physiol. Lung Cell. Mol. Physiol. 307, L576–L585. 10.1152/ajplung.00162.2014 25128524 PMC4187037

[B52] Penumatsa K. C. Toksoz D. Warburton R. R. Kharnaf M. Preston I. R. Kapur N. K.. (2017). Transglutaminase 2 in pulmonary and cardiac tissue remodeling in experimental pulmonary hypertension. Am. J. Physiol. Lung Cell. Mol. Physiol. 313, L752–Ll762. 10.1152/ajplung.00170.2017 28775095 PMC5792178

[B53] Pitsiou G. Papakosta D. Bouros D. (2011). Pulmonary hypertension in idiopathic pulmonary fibrosis: a review. Respiration 82, 294–304. 10.1159/000327918 21677422

[B54] Poor H. D. Girgis R. Studer S. M. (2012). World Health Organization Group III pulmonary hypertension. Prog. Cardiovasc. Dis. 55, 119–127. 10.1016/j.pcad.2012.08.003 23009908

[B55] Ricard N. Tu L. Le Hiress M. Huertas A. Phan C. Thuillet R.. (2014). Increased pericyte coverage mediated by endothelial-derived fibroblast growth factor-2 and interleukin-6 is a source of smooth muscle-like cells in pulmonary hypertension. Circulation 129, 1586–1597. 10.1161/CIRCULATIONAHA.113.007469 24481949

[B56] Ruggiero R. M. Bartolome S. Torres F. (2012). Pulmonary hypertension in parenchymal lung disease. Heart Fail. Clin. 8, 461–474. 10.1016/j.hfc.2012.04.010 22748906

[B57] Saadjian A. Y. Paganelli F. Gaubert M. L. Levy S. Guieu R. P. (1999). Adenosine plasma concentration in pulmonary hypertension. Cardiovasc. Res. 43, 228–236. 10.1016/S0008-6363(99)00059-0 10536708

[B58] Schroll S. Arzt M. Sebah D. Nüchterlein M. Blumberg F. Pfeifer M. (2010). Improvement of bleomycin-induced pulmonary hypertension and pulmonary fibrosis by the endothelin receptor antagonist Bosentan. Respir. Physiol. Neurobiol. 170, 32–36. 10.1016/j.resp.2009.11.005 19931426

[B59] Schroll S. Lange T. J. Arzt M. Sebah D. Nowrotek A. Lehmann H.. (2013). Effects of simvastatin on pulmonary fibrosis, pulmonary hypertension and exercise capacity in bleomycin-treated rats. Acta Physiol. 208, 191–201. 10.1111/apha.12085 23527830

[B60] Song A. Zhang Y. Han L. Yegutkin G. G. Liu H. Sun K.. (2017). Erythrocytes retain hypoxic adenosine response for faster acclimatization upon re-ascent. Nat. Commun. 8:14108. 10.1038/ncomms14108 28169986 PMC5309698

[B61] Sun C. X. Zhong H. Mohsenin A. Morschl E. Chunn J. L. Molina J. G.. (2006). Role of A2B adenosine receptor signaling in adenosine-dependent pulmonary inflammation and injury. J. Clin. Invest. 116, 2173–2182. 10.1172/JCI27303 16841096 PMC1501110

[B62] Sun K. Liu H. Song A. Manalo J. M. D'alessandro A. Hansen K. C.. (2017). Erythrocyte purinergic signaling components underlie hypoxia adaptation. J. Appl. Physiol. 123, 951–956. 10.1152/japplphysiol.00155.2017 28572494 PMC5668449

[B63] Synnestvedt K. Furuta G. T. Comerford K. M. Louis N. Karhausen J. Eltzschig H. K.. (2002). Ecto-5'-nucleotidase (CD73) regulation by hypoxia-inducible factor-1 mediates permeability changes in intestinal epithelia. J. Clin. Invest. 110, 993–1002. 10.1172/JCI0215337 12370277 PMC151145

[B64] Toldo S. Zhong H. Mezzaroma E. Van Tassell B. W. Kannan H. Zeng D.. (2012). GS-6201, a selective blocker of the A2B adenosine receptor, attenuates cardiac remodeling after acute myocardial infarction in the mouse. J. Pharmacol. Exp. Ther. 343, 587–595. 10.1124/jpet.111.191288 22923737 PMC11047795

[B65] Van Linden A. Eltzschig H. K. (2007). Role of pulmonary adenosine during hypoxia: extracellular generation, signaling and metabolism by surface adenosine deaminase/CD26. Expert Opin. Biol. Ther. 7, 1437–1447. 10.1517/14712598.7.9.1437 17727332

[B66] Van Rheen Z. Fattman C. Domarski S. Majka S. Klemm D. Stenmark K. R.. (2011). Lung extracellular superoxide dismutase overexpression lessens bleomycin-induced pulmonary hypertension and vascular remodeling. Am. J. Respir. Cell Mol. Biol. 44, 500–508. 10.1165/rcmb.2010-0065OC 20539010 PMC3095923

[B67] Ventetuolo C. E. Klinger J. R. (2012). WHO Group 1 pulmonary arterial hypertension: current and investigative therapies. Prog. Cardiovasc. Dis. 55, 89–103. 10.1016/j.pcad.2012.07.002 23009906

[B68] Volmer J. B. Thompson L. F. Blackburn M. R. (2006). Ecto-5′-nucleotidase (CD73)-mediated adenosine production is tissue protective in a model of bleomycin-induced lung injury. J. Immunol. 176, 4449–4458. 10.4049/jimmunol.176.7.4449 16547283

[B69] Wakamiya M. Blackburn M. R. Jurecic R. Mcarthur M. J. Geske R. S. Cartwright J.. (1995). Disruption of the adenosine-deaminase gene causes hepatocellular impairment and perinatal lethality in mice. Proc. Natl. Acad. Sci. U.S.A. 92, 3673–3677. 10.1073/pnas.92.9.3673 7731963 PMC42023

[B70] Wirsdörfer F. De Leve S. Cappuccini F. Eldh T. Meyer A. V. Gau E.. (2016). Extracellular adenosine production by ecto-5′-nucleotidase (CD73) enhances radiation-induced lung fibrosis. Cancer Res. 76, 3045–3056. 10.1158/0008-5472.CAN-15-2310 26921334 PMC4960984

[B71] Zhou Y. Murthy J. N. Zeng D. Belardinelli L. Blackburn M. R. (2010). Alterations in adenosine metabolism and signaling in patients with chronic obstructive pulmonary disease and idiopathic pulmonary fibrosis. PLoS ONE 5:e9224. 10.1371/journal.pone.0009224 20169073 PMC2821921

[B72] Zhou Y. Schneider D. J. Blackburn M. R. (2009). Adenosine signaling and the regulation of chronic lung disease. Pharmacol. Ther. 123, 105–116. 10.1016/j.pharmthera.2009.04.003 19426761 PMC2743314

[B73] Zimmerman M. A. Grenz A. Tak E. Kaplan M. Ridyard D. Brodsky K. S.. (2013). Signaling through hepatocellular A2B adenosine receptors dampens ischemia and reperfusion injury of the liver. Proc. Natl. Acad. Sci. U.S.A. 110, 12012–12017. 10.1073/pnas.1221733110 23812746 PMC3718151

