# Supplementary material for: Switching-Off Adora2b in Vascular Smooth Muscle Cells Halts the Development of Pulmonary Hypertension
Source: Front Physiol. 2018 Jun 1;9:555. doi: 10.3389/fphys.2018.00555 (PMC5992271; doi:10.3389/fphys.2018.00555)
Supplement: Supplementary file 1 [file Image_1.pdf]

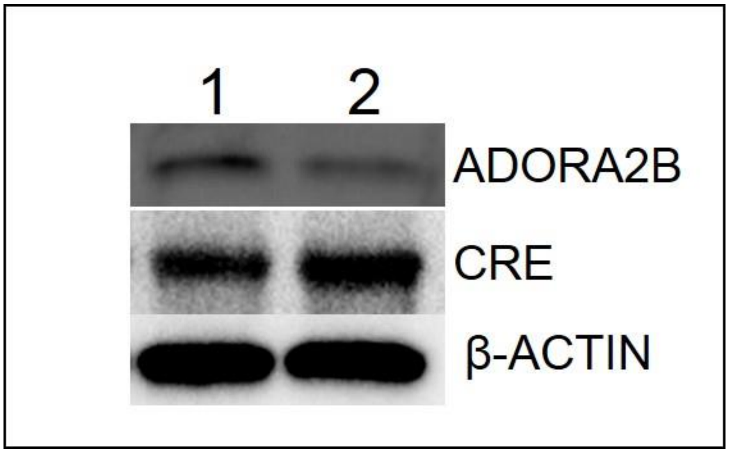

Supplementary Figure 1

Protein levels of Adora2b, CRE and βactin from pulmonary artery smooth muscle cells (PASMCs) isolated from a $Tagln^{Cre}$ mouse (Lane 1) or an $Adora2b^{f/f}$- $Tagln^{Cre}$ mouse (Lane 2).

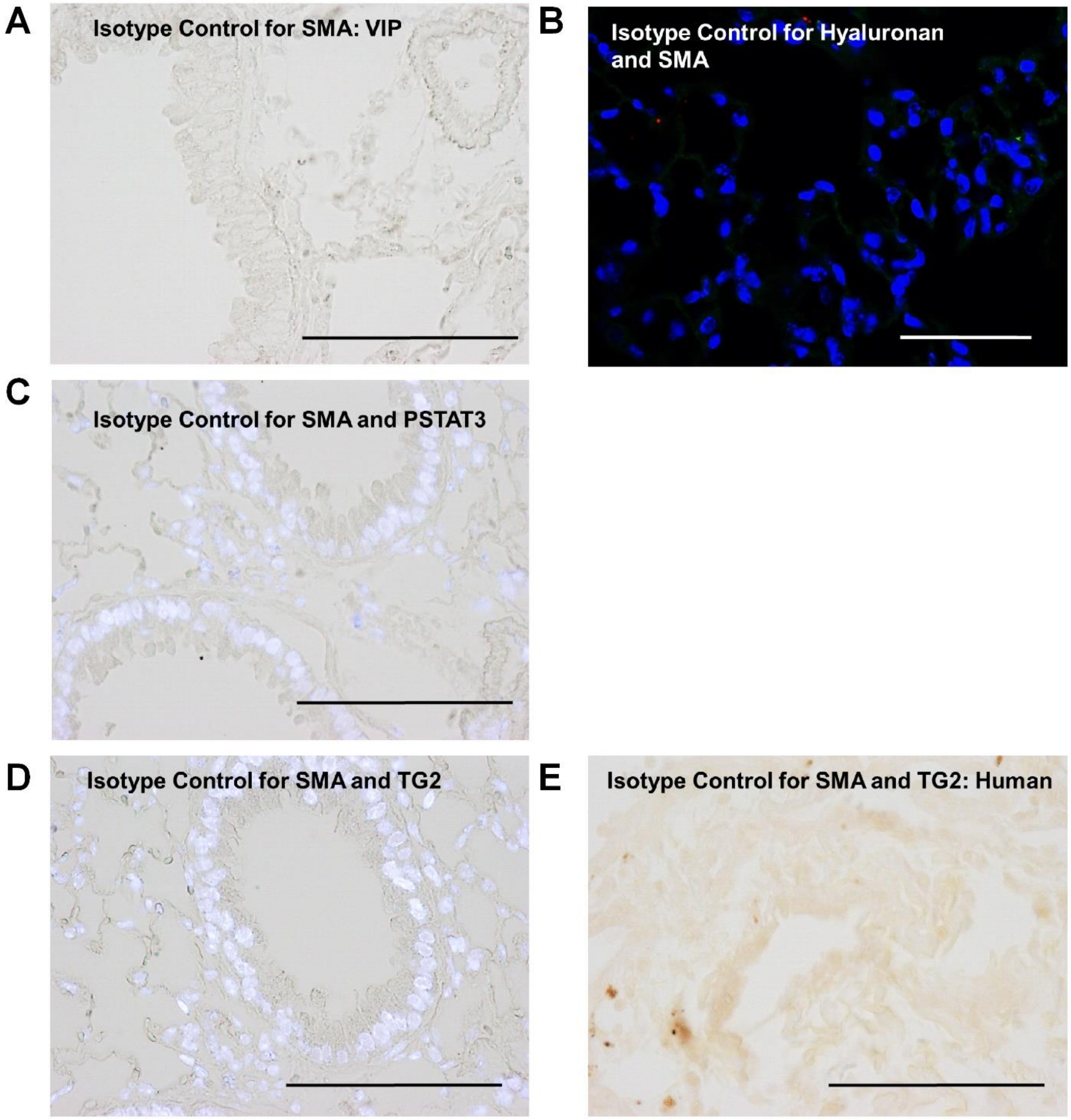

Supplementary Figure 2

Isotype control for mouse immuno-histochemistry (IHC) for alpha smooth muscle actin (αSMA) using VIP development (**A**); isotype control immuno fluorescence for αSMA and hyaluronan (**B**); isotype control IHC staining for αSMA (Vector Blue) and PSTAT3 (DAB) (**C**); isotype control IHC staining for αSMA (Vector Blue) and Tg2 (Vector Red) (**D**). Isotype control for human IHC staining for human αSMA (BCIP/NBT) and Tg2 (Vector Red) (**E)**.

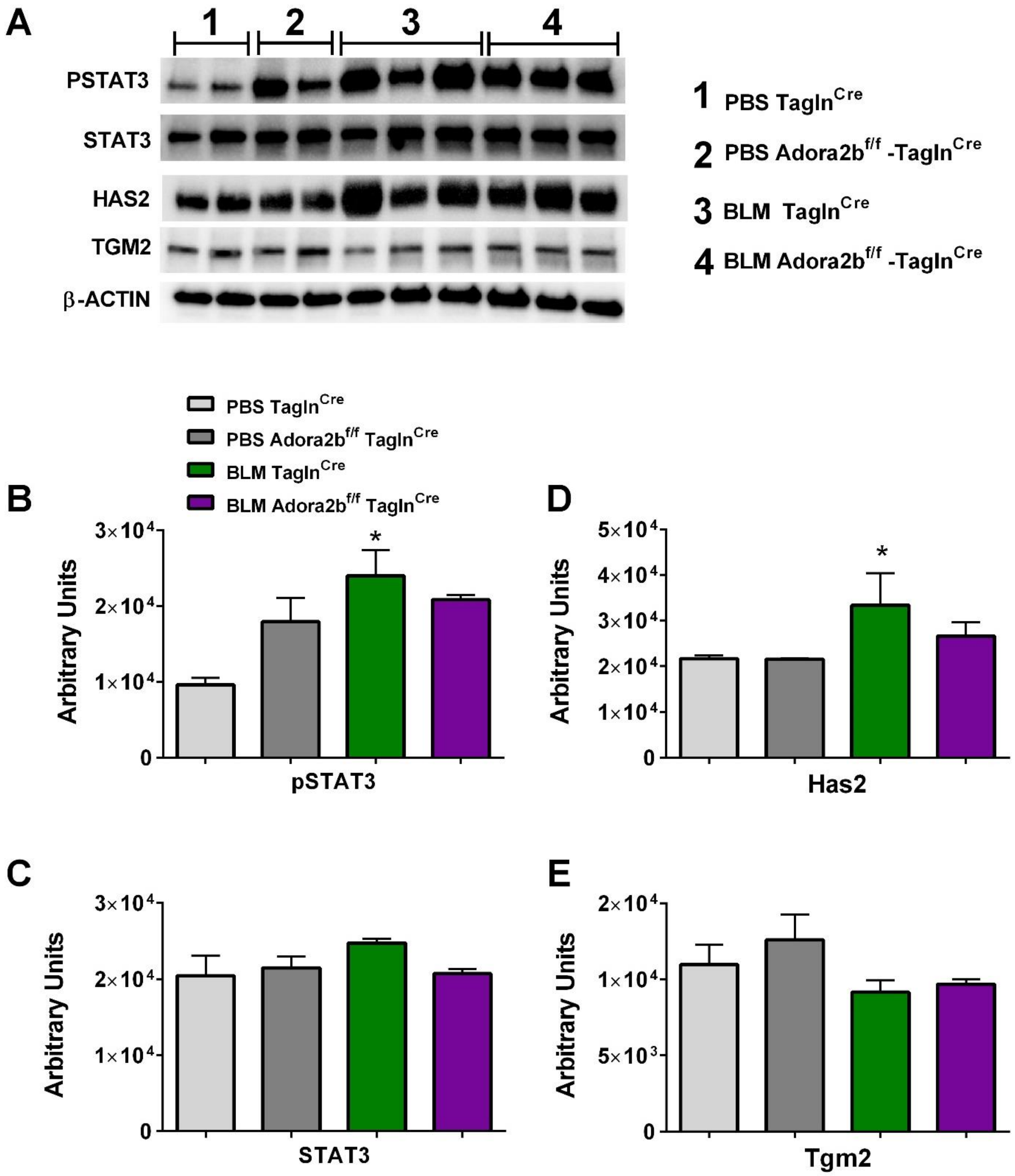

Supplementary Figure 3

Western blot for PSTAT3, STAT3, Has2, Tgm2 and β-actin for PBS or BLM exposed Tagln^{Cre} and Adora2b$^{f/f}$-Tagln^{Cre} mice. Lane 1 represents PBS Tagln^{Cre}, lane 2 represents PBS Adora2b$^{f/f}$-Tagln^{Cre}, lane 3 represents BLM Tagln^{Cre} and lane 4 represents BLM Adora2b$^{f/f}$-Tagln^{Cre} groups. Densitometry data for PSTAT3 **(B)**, STAT3 **(C)**, Has2 **(D)** and Tgm2 **(E)**. : *$P < 0.05$ refer to

comparisons between Adora2b-Tagln^{Cre} BLM and Tagln^{Cre} BLM treatment groups N=2 for PBS and N=3 for BLM groups.
