# Supplementary material for: Switching-Off Adora2b in Vascular Smooth Muscle Cells Halts the Development of Pulmonary Hypertension
Source: Front Physiol. 2018 Jun 1;9:555. doi: 10.3389/fphys.2018.00555 (PMC5992271; doi:10.3389/fphys.2018.00555)
Supplement: Supplementary file 2 [file Table_1.DOCX]

**Supplementary Table 1.** Clinical Data from the specimens to determine adenosine receptors by western blots

|  | **Controls** | **PAH** |
| --- | --- | --- |
|  | n=7 | n=8* |
| **Age, yrs** | 56 ± 4.3 | 33 ± 5.1 |
| **Sex, M/F (ratio)** | 4 / 3 | 3 / 5 |
| **Mutation in *BMPR2* gene, n** |  |  |
| Carrier | NA | 0 |
| No-carrier | NA | 8 |
|  |  |  |
| **NYHA functional class, n** |  |  |
| class II | NA | 2 |
| class III | NA | 4 |
| class IV | NA | 2 |
| **mPAP, mmHg** | NA | 75.14 ± 11.3 |
| **CI, l/min/m²** | NA | 4.1 ± 0.7 |
| **PVRi, mmHg/l/min/m²** | NA | 10.6 ± 1.4 |
| **PCWP, mmHg** | NA | 8 ± 0.5 |
|  |  |  |
| NYHA=New York Heart Association; mPAP=mean pulmonary artery pressure; CI=cardiac index; PVRi= pulmonary vascular resistance index; PCWP=pulmonary capillary wedge pressure; ±=SEM *clinical data for one PAH was not found | | |
|  |  |  |
|  |  |  |
|  |  |  |
|  |  |  |
|  |  |  |
