# Supplementary material for: Switching-Off Adora2b in Vascular Smooth Muscle Cells Halts the Development of Pulmonary Hypertension
Source: Front Physiol. 2018 Jun 1;9:555. doi: 10.3389/fphys.2018.00555 (PMC5992271; doi:10.3389/fphys.2018.00555)
Supplement: Supplementary file 3 [file Table_2.DOCX]

**Supplementary Table 2.** List of primary antibodies used.

| αSMA | A5228 Sigma Aldrich (St Louis, MO)  (IHC 1:1000 human, mouse)  F3777 – FITC conjugated Sigma Aldrich (St Louis, MO)  (IHC 1:1000 human, mouse) |
| --- | --- |
| β-ACTIN | 49675 Cell Signalling (Danvers, MA)  (WB 1:1000 human, mouse) |
| ADORA2A | PA1-042 Thermo Fisher (Waltham, MA)  (WB: 1:1000) |
| ADORA2B | NBP2-41312 NOVUS Biologicals (Littleton, CO)  (WB: 1:1000 human)  Sc-28996 Santa Cruz Biotechnology (Dallas, TX)  (WB: 1:200 mouse) |
| FN |  |
| GAPDH | AM4300 Themo Fisher Scientific (WB 1:1000) |
| HAS2 | Sc-34068 Santa Cruz Biotechnology (Dallas, TX)  (WB:1:500 mouse) |
| Hyaluronan | #385911 CalBioChem (San Diego, CA) (1:125 IHC) |
| STAT3 | 9132 Cell Signalling (Danvers, MA)  (WB: 1:1000 mouse) |
| PSTAT3 (S727) | Ab30647 Abcam (Cambridge, MA)  (IHC 1:100 mouse) |
| PSTAT3 (Y703) | Ab76315 Abcam (Cambridge, MA)  (WB: 1:2000 mouse) |
| Rabbit Secondary | 7074S Cell Signalling (Danvers, MA)  (WB: 1:1000) |
| TGM2 | GTX111701 Genetex (Irvine, CA)  (IHC: 1:200 mouse, human)  (WB:1:1000 mouse) |
