# Supplementary material for: Switching-Off Adora2b in Vascular Smooth Muscle Cells Halts the Development of Pulmonary Hypertension
Source: Front Physiol. 2018 Jun 1;9:555. doi: 10.3389/fphys.2018.00555 (PMC5992271; doi:10.3389/fphys.2018.00555)
Supplement: Supplementary file 4 [file Table_3.DOCX]

**Supplementary Table 3.** List of primers used

| Gene | Primer Forward | Primer Reverse |
| --- | --- | --- |
| 18srRNA | GTAACCCGTTGAACCCCATT | CCATCCAATCGGTAGTAGCG |
| HUMAN |  |  |
| ADORA1 | TGCACTGACTTCTACGGCTG | GGTCCCCGTGACCAAACTT |
| ADORA2A | CGCTCCGGTACAATGGCTT | TTGTTCCAACCTAGCATGGGA |
| ADORA2B | TGCACTGACTTCTACGGCTG | GGTCCCCGTGACCAAACTT |
| ADORA3 | TCATTCTACTCTCCTTGGCTCTC | GTGGGCATTGTAGTTGCAGAT |
| CD39 | AGGTGCCTATGGCTGGATTAC | CCAAAGCTCCAAAGGTTTCCT |
| CD73 | CCAGTACCAGGGCACTATCTG | TGGCTCGATCAGTCCTTCCA |
| ADA | GGGCTGCTGAACGTCATTG | AGGCATGTAGTAGTCAAACTTGG |
| HAS2 | TCCAAAGAGTGTGGTTCCAA | GACAGGCTGAGGACGACTTT |
| IL6 | AATTCGGTACATCCTCGACGG | TTGGAAGGTTCAGGTTGTTTTCT |
| TGM2 | GCACCTTGATGAGGTTGGAC | CAACCTGGAGCCTTTCTCTG |
| MOUSE |  |  |
| Adora1 | TGTGCCCGGAAATGTACTGG | TCTGTGGCCCAATGTTGATAAG |
| Adora2a | TTCCACTCCGGTACAATGGC | CGATGGCGAATGACAGCAC |
| Adora2b |  |  |
| Adora3 | AAGTAAGAACGGTGGCCCTC | TCCCTGCCTTCCCATTAACC |
| CD39 | AAGGTGAAGAGATTTTGCTCCAA | TTTGTTCTGGGTCAGTCCCAC |
| CD73 | GGACATTTGACCTCGTCCAAT | GGGCACTCGACACTTGGTG |
| ADA | AGAGGATCGCCTACGAGTTTG | TTGGGTCCACCTTGGAATTGG |
| Col1a1 | GGTTTCCACGTCTCACCATT | CGGCTCCTGCTCCTCTTAG |
| Fn | ACTGGATGGGGTGGGAAT | GGAGTGGCACTGTCAACCTC |
| Has2 | ACAGATGAGGCAGGGTCAAG | TGGGGTGGAAAGAGAGAAGT |
| IL6 | TAGTCCTTCCTACCCCAATTTCC | TTGGTCCTTAGCCACTCCTTC |
| Tgm2 | GACAATGTGGAGGAGGGATCT | CTCTAGGCTGAGACGGTACAG |
